# Genome reorganization and its functional impact during breast cancer progression

**DOI:** 10.7554/eLife.108135

**Published:** 2026-06-16

**Authors:** Kathleen S Metz Reed, Andrew Fritz, Haley Greenyer, Kerstin Heselmeyer-Haddad, Seth Frietze, Janet Stein, Gary Stein, Tom Misteli

**Affiliations:** 1 National Cancer Institute, National Institutes of Health Bethesda United States; 2 Department of Biochemistry and University of Vermont Cancer Center, University of Vermont Larner College of Medicine Burlington United States; 3 OMICS Technology Facility, Genetics Branch, Center for Cancer Research, National Cancer Institute, National Institutes of Health Bethesda United States; 4 College of Nursing and Health Sciences, University of Vermont Cancer Center Burlington United States; Institute of Radiation Medicine, Chinese Academy of Medical Sciences and Peking Union Medical College China; Shengjing Hospital of China Medical University China

**Keywords:** genome organization, chromatin loops, epigenetics, breast cancer, enhancers, MCF10, Human

## Abstract

Cancer progression involves extensive alterations in epigenetic and gene expression programs, but the accompanying changes in higher-order genome organization remain less well understood. Using high-resolution Micro-C mapping in the MCF10 cell model of breast cancer, we profiled chromatin compartments, topologically associated domains, and chromatin loops. We find large-scale compartmental shifts occur predominantly in early stages of cancer development, with more fine-scale structural changes in topologically associating domains and loops accumulating during the later transition to metastasis. Relating these chromatin features to gene expression and enhancer-associated histone marks revealed that many differentially expressed genes are physically connected to distal regulatory elements. While enhancer–promoter contact frequency and distal enhancer activity correlated with gene expression, strong changes in chromatin looping were relatively infrequent during progression, suggesting that alterations in chromatin contacts are not globally necessary, but may facilitate gene regulation at a subset of genes. These results elucidate the connection between gene regulation and genome remodeling in a cell-based cancer progression model.

## Introduction

The eukaryotic genome is highly organized in the cell nucleus. Among the most prominent structural features are kilobase-sized chromatin loops, medium-scale topologically associating domains (TADs), and higher-order compartments (Dekker and Mirny, 2016; Jerkovic´ and Cavalli, 2021; Misteli, 2020; Pombo and Dillon, 2015; Rowley and Corces, 2018). How chromatin organization contributes to epigenetic control of gene regulation, including in physiological and pathological settings, such as cancer, remains only partially understood (Corces et al., 2016; Peng et al., 2022; Zhang et al., 2021; Amodeo et al., 2025).

A prominent mechanism to generate higher-order chromatin structures is loop extrusion (Davidson and Peters, 2021; de Wit and Nora, 2023; Sanborn et al., 2015). During this process, the multi-component cohesin complex is loaded onto chromatin and, using its intrinsic molecular motor activity, extrudes chromatin bidirectionally along the genome to form a loop or a domain until it encounters the major chromatin architectural protein CTCF bound to convergently oriented binding sites. The encounter of cohesin with bound CTCF stalls the extrusion process and generates a chromatin loop or TAD. While loop extrusion has universally been implicated in the formation of chromatin loops and domains, the formation of chromatin features by other mechanisms has also been observed, especially at a smaller scale (Goel et al., 2023; Hsieh et al., 2020; Oberbeckmann et al., 2024; Rowley and Corces, 2018).

A common property of higher-order chromatin folding is that the resulting loops, domains, and compartments bring distal genome elements into spatial proximity which has been implicated in gene regulation (Jerkovic´ and Cavalli, 2021). For example, it has been suggested that one function of TADs is to facilitate the interaction of regulatory elements, particularly gene enhancers, with their target genes located within the same TAD (Chakraborty et al., 2024; Chakraborty et al., 2025; da Costa-Nunes and Noordermeer, 2023; Xiao et al., 2021; Sood and Misteli, 2022). Similarly, long-range interactions via chromatin loops are thought to be essential at some loci to bring gene enhancers into proximity to their target promoters (D’Ippolito et al., 2018; Kane et al., 2022; Schoenfelder and Fraser, 2019; Stolper et al., 2023; Popay and Dixon, 2022). However, regulation of many gene loci also appears independent of chromatin organization, and enhancer–promoter proximity often does not correlate with gene activity (Benabdallah et al., 2019; Despang et al., 2019; Hansen et al., 2024; Hsieh et al., 2022). In fact, acute depletion of cohesin revealed genome-wide disruption of chromatin organization but surprisingly limited impact on gene expression (Rao et al., 2017). A possible explanation for these divergent findings is that chromatin organization may be functionally more relevant to bring about changes in gene activity rather than maintenance of gene expression, as suggested by several cohesin depletion studies (Hsieh et al., 2022; Cuartero et al., 2018; Popay et al., 2024).

Genome organization is likely relevant for cancer and its progression. Mutations in loop extrusion machinery, such as cohesin and the cohesin processivity factor NIPBL or at CTCF-binding sites have been reported in many cancers (Corces et al., 2016; Peng et al., 2022; Wang et al., 2022; Guo et al., 2018; Katainen et al., 2015). In addition, many structural variants (SVs) such as deletions, duplications, and translocations have been documented in various cancer subtypes where SVs and the ensuing reorganization around them can lead to aberrant gene regulation (Cosenza et al., 2022; Mitelman et al., 2007; Della Chiara et al., 2023).

Despite these observations, the full extent of genome reorganization during cancer progression and its functional consequences remains largely unknown. Several studies primarily focused on large-scale reorganizations have found changes in higher-order chromatin organization such as chromosome clustering and dynamic compartments, some of which correlated with changes in differentially expressed oncogenes and enhancers (Barutcu et al., 2015; Fritz et al., 2018; Johnstone et al., 2020; Li et al., 2021; Mourad et al., 2014; Rickman et al., 2012; Taberlay et al., 2016; Yang et al., 2020; Zhou et al., 2019). Analysis of TADs in various cancers has found mixed results, with some studies pointing to increased TADs and gained boundaries and others observing more stable TAD organization or weakened boundaries (Johnstone et al., 2020; Taberlay et al., 2016; Yang et al., 2020; Kim et al., 2022; Rossini et al., 2024). Furthermore, cancer-associated SVs, such as chromosomal translocations, have been related to altered gene expression, for example via enhancer-hijacking (Mortenson et al., 2024; Wang et al., 2021). However, how local chromatin loops and TADs are restructured during oncogenic reprogramming and how these changes relate to cancer-associated gene expression has not been well documented.

We address the question of how local and global chromatin organization changes during cancer progression and how these events relate to cancer gene expression programs by generating high-resolution Micro-C maps in the well-established MCF10 breast cancer progression model (Puleo and Polyak, 2021). This cancer model consists of three epithelial cell lines that were sequentially derived from a non-cancerous individual (Soule et al., 1990). MCF10A is an adherent epithelial non-cancerous cell line that spontaneously immortalized from the initial sample. Pre-malignant MCF10AT1 cells were derived from MCF10A cells by overexpressing mutant Ha-Ras oncogene followed by long-term passage as mouse xenografts (Dawson et al., 1996). In immunocompromised mice, MCF10AT1 cells form precancerous lesions, and approximately 25% progress to invasive carcinoma over time. The MCF10CA1a cell line is derived from metastatic tumors generated from xenografted MCF10AT1 cells and is considered fully malignant, metastatic, and highly aggressive, forming tumors in 100% of xenografted mice which can metastasize rapidly to the lung (Santner et al., 2001). The MCF10 progression series represents a spectrum of cells that share a similar genetic background but are increasingly more cancerous, and they have been widely used to study the genetic and epigenetic changes that occur during cancer progression and epithelial–mesenchymal transition (Fritz et al., 2018; Bhardwaj et al., 2017; Buchanan et al., 2007; Dorgham et al., 2023; Kadota et al., 2010; Lin et al., 2019; Maguire et al., 2016; Marella et al., 2009; Rhee et al., 2008; So et al., 2012).

In this study, comparing fine-scale chromatin organization and other epigenetic features in the MCF10 cancer progression model has allowed us to identify changes in genome reorganization and relate them to changes in gene expression, including of cancer progression-associated genes. Our results provide novel insights into the principles of chromatin-mediated gene regulation and into the dynamic structure–function relationship contributing to genome regulation derived from an in vitro cancer progression model.

## Results

### Mapping global and local genome organization across breast cancer progression

**Figure 1.**
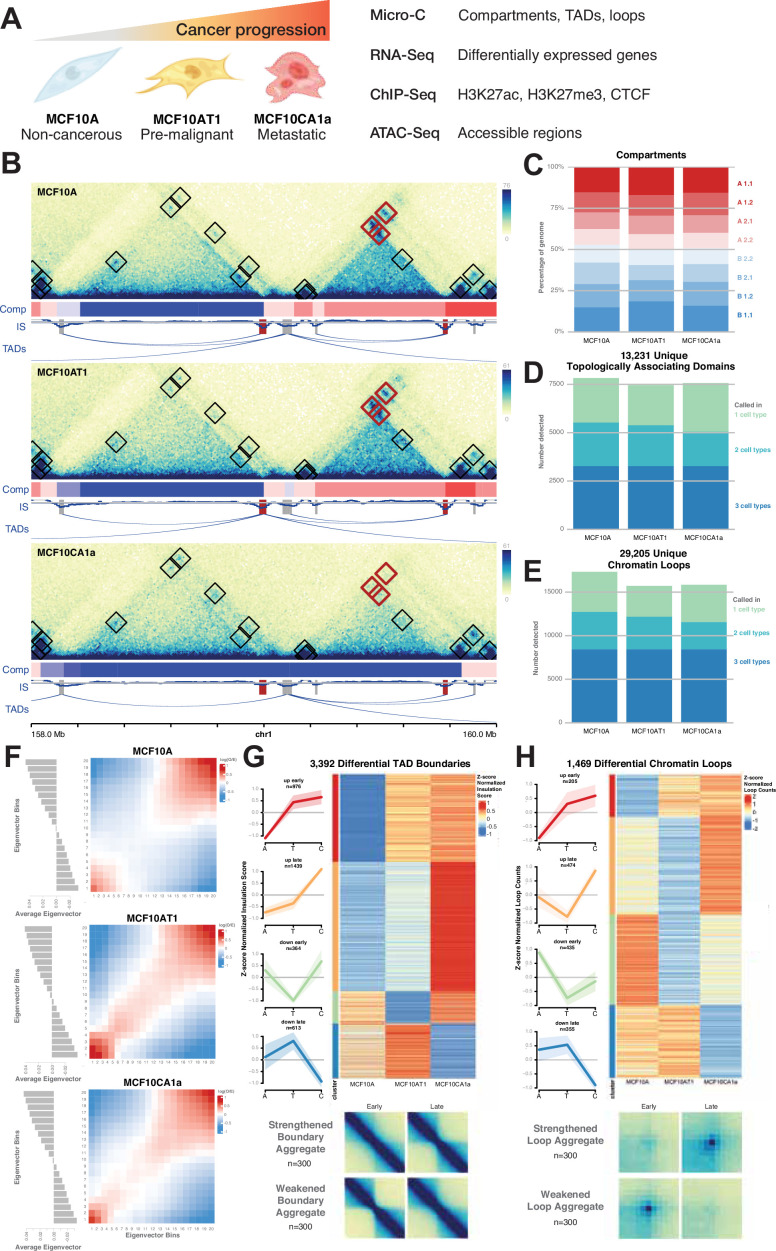
Reorganization of compartments, topologically associating domains (TADs), and loops during breast cancer progression. (**A**) A diagram of the experimental design. Three epithelial cell lines represent various stages of breast cancer progression; MCF10A are non-cancerous, MCF10AT1 are pre-malignant, and MCF10CA1a are metastatic. In each cell line, we generated 5-kb resolution Micro-C to identify features such as compartments, TADs, and chromatin loops. We overlapped these features with functional changes in gene expression from RNA-Seq, histone modifications, and CTCF binding from ChIP-Seq, and chromatin accessibility from ATAC-Seq. (**B**) Micro-C maps of a 2-Mb region of chromosome 1 in MCF10A (non-cancerous), MCF10AT1 (pre-cancerous), and MCF10CA1a (metastatic) cells at 5-kb resolution. Each map has annotations for loop calls, both static (black boxes) and differential (red boxes). Below each map is a track indicating compartment calls from CALDER (dark red is most A-like, dark blue is most B-like) as well as insulation scores tracks with static (gray) and differential (red) boundaries marked. Ribbons indicate TAD calls for each cell type. (**C**) Lengths of the genome assigned to each compartment in each cell type. (**D**) Numbers of TAD and (**E**) loop calls from each cell type, colored by the number of cell types in which they were initially detected . (**F**) Saddle plots of interactions between regions of different compartments in MCF10A, MCF10AT1, and MCF10CA1a. Bottom plots indicate the average eigenvector value for each compartment ventile. Plots shown are for chromosomes 2, 12, and 17 (see Methods). (**G**) Left: Differential TAD boundaries clustered by their timing of change, depicted in line plots and heatmap. Right: Aggregate plots of weakened and strengthened TAD boundaries (*n* = 100). (**H**) Left: Differential chromatin loops clustered by their timing of change, depicted in line plots and heatmap. Right: Aggregate plots of weakened and strengthened loops (*n* = 100).

To understand how genome organization changes during cancer progression, we generated high-resolution (5 kb) genome-wide maps of chromatin contacts using Micro-C in the MCF10A, MCF10AT1, and MCF10CA1a cancer progression series (Figure 1A, Figure 1—figure supplement 1A). We obtained high-quality data with at least 1 billion Micro-C contacts per cell line, spread across two biological replicates with four technical replicates each (Supplementary file 1).

**Figure 1—figure supplement 1.**
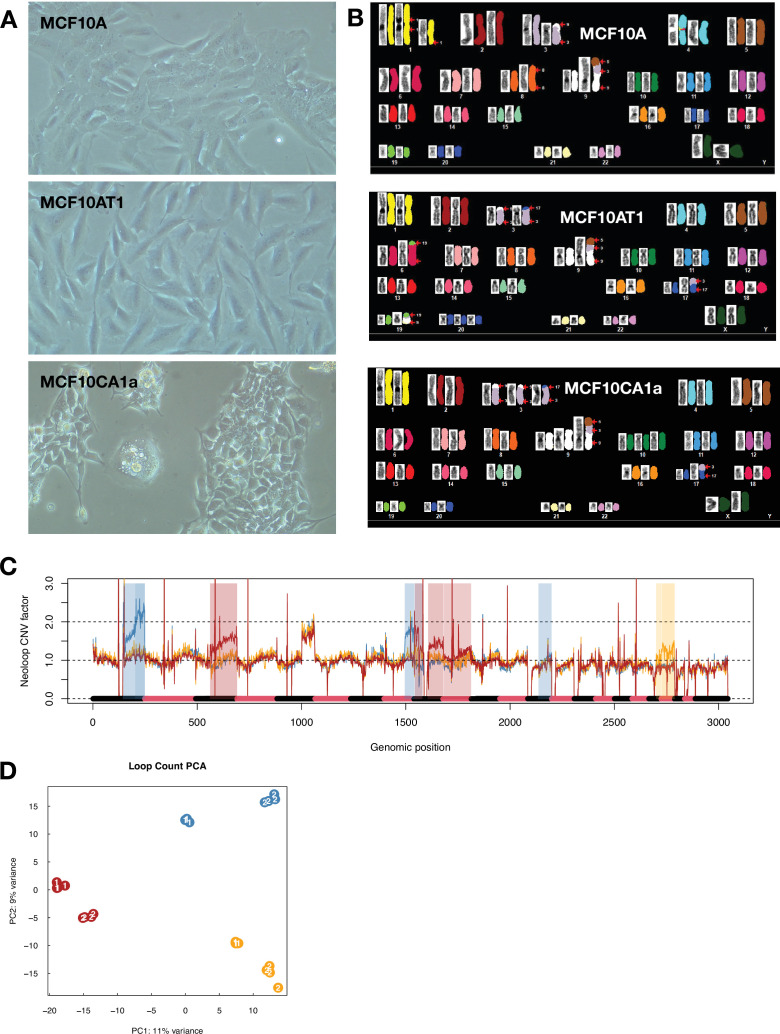
Karyotypic analysis and Micro-C loop reproducibility. (**A**) Brightfield microscopy images of MCF10A, MCF10AT1, and MCF10CA1a cell lines. (**B**) Representative SKY karyotype example images from MCF10A, MCF10AT1, and MCF10CA1a cell lines. (**C**) Copy number variation (CNV) factors for loops across the genome as generated from NeoloopFinder. Highlighted regions indicate areas of karyotypic differences that were corrected for in the identification of differential loops; blue is regions with higher CNV in MCF10A, yellow is regions with higher CNV in MCF10AT1, and red is regions with higher CNV in MCF10CA1a. Regions that have shared karyotypic abnormalities among all three cell lines, such as the q arm of chromosome 5 which is duplicated and translocated to chromosome 9 in all cell types, did not require correction. (**D**) Micro-C chromatin loop count principal component analysis (PCA). Blue circles indicate MCF10A samples, yellow circles indicate MCF10AT1, and red circles indicate MCF10CA1a.

**Figure 1—figure supplement 2.**
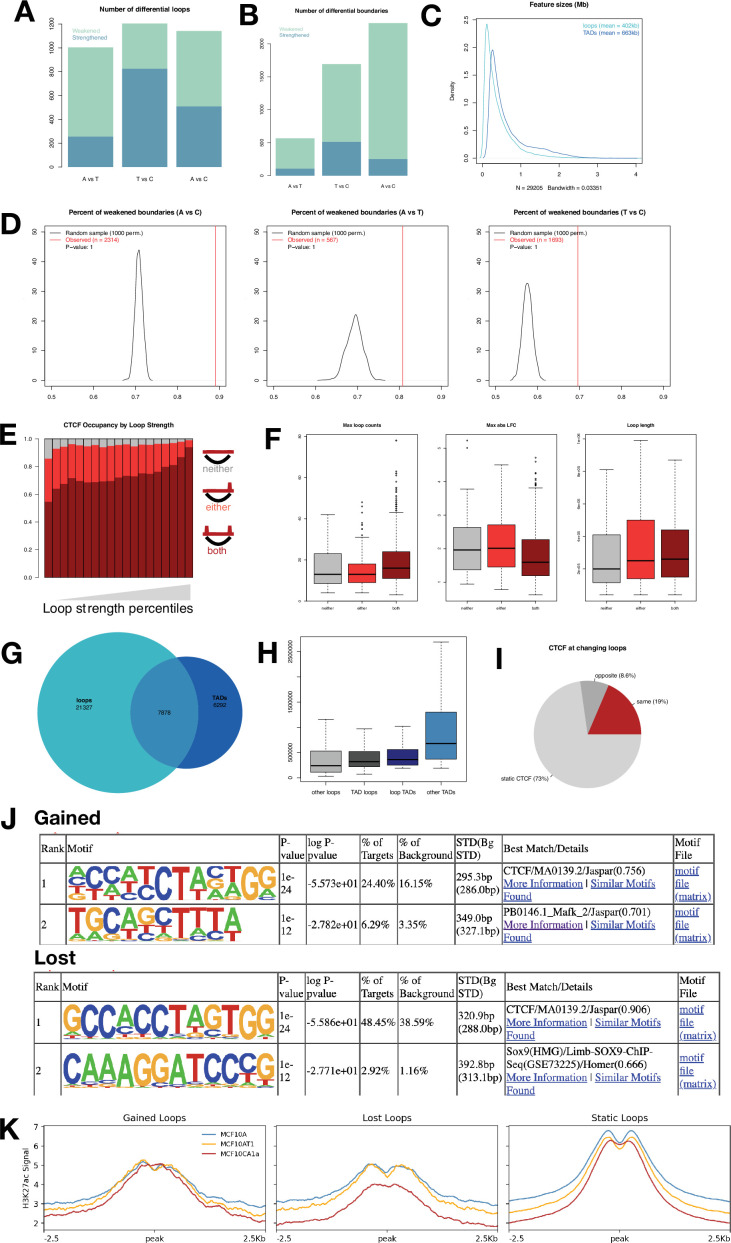
Differential loop and topologically associating domain (TAD) feature details. (**A**) The number of chromatin loops that exhibit a significant increase (blue) or decrease (green) in contact frequency between each pairwise comparison of cell types. (**B**) The number of TAD boundaries that exhibit a significant increase (blue) or decrease (green) in insulation score between each pairwise comparison of cell types. (**C**) Size distributions of chromatin loops and TADs. (**D**) Permutation test results of weakened boundaries observed in each pairwise comparison as compared to a random sampling. (**E**) Percent of loops with one or both anchors overlapping CTCF ChIP-Seq peaks based on the strength of the loop (20 bins). (**F**) Boxplots of (left to right) maximum loop Micro-C counts, maximum loop count log_2_ fold-change, and loop length based on loop-CTCF class; neither (gray), one anchor overlap (light red), or both anchors overlap (dark red). (**G**) Venn diagram showing the degree of overlap between chromatin loops and TADs. (**H**) Feature sizes of loops that do not overlap TADs (light gray), loops that do overlap TADs (dark gray), TADs that do overlap loops (dark blue), and TADs that do not overlap loops (light blue). (**I**) Pie chart of the percentage of differential loops that have a correlated change in CTCF binding at either anchor; light gray indicates static CTCF peaks, dark gray indicates CTCF peaks that change in the opposite way as the loop (i.e. a strengthened loop with loss of CTCF binding), and dark red indicates CTCF peaks that change in the same way as the loop (i.e. a strengthened loop with gain of CTCF binding). (**J**) De novo motif results from HOMER for ATAC-Seq peaks within the anchors of gained/strengthened (top) and lost/weakened (bottom) chromatin loops. (**K**) H3K27ac ChIP-Seq aggregate profiles at the anchors of gained/strengthened, lost/weakened, and static loops.

**Figure 1—figure supplement 3.**
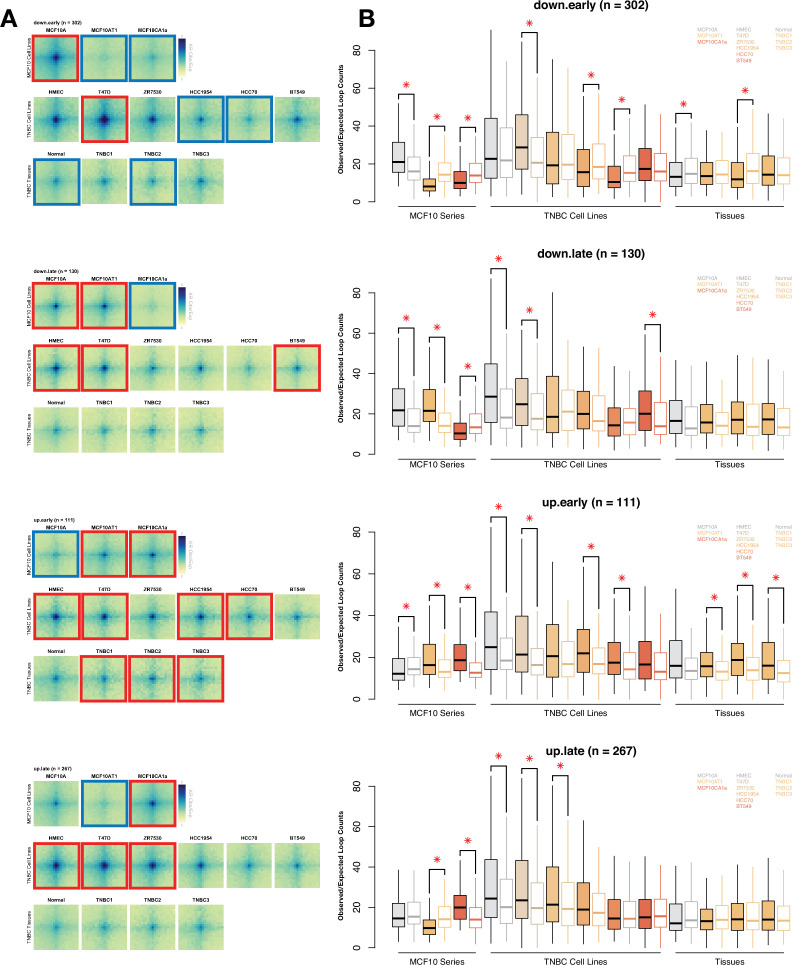
Differential MCF10 loops are conserved in cancer cell lines. (**A**) Aggregate loop analysis of chromatin loops from each differential cluster as found in the MCF10 series compared to triple negative breast cancer (TNBC) cell lines and primary patient tissues (Kim et al., 2022). Red boxes indicate loops that have significantly higher observed/expected contact frequency among loops in that cluster as compared to a random sampling of an equal number of chromatin loops that are static in the MCF10 series, as determined in (**B**). (**B**) Boxplots representing quantifications of the aggregate plots shown in (**A**). Filled boxes indicate the counts from loops in each differential cluster, while empty boxes indicate a matched sample of static loops that are of similar size. Stars indicate cell lines where there is a significant difference between the counts of the given loops and the static matched set (*P* ≤ 0.05, Wilcoxon rank sum test).

**Figure 1—figure supplement 4.**
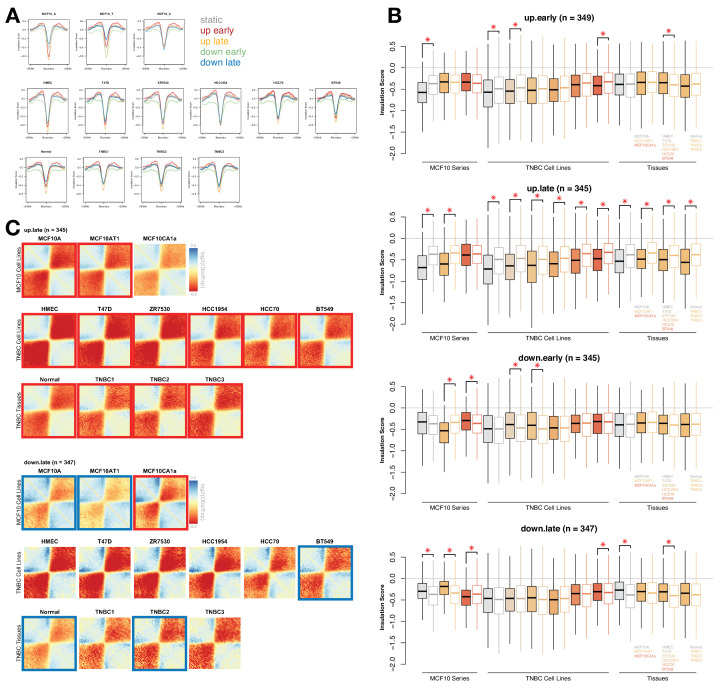
Differential topologically associating domain (TAD) boundaries are conserved in cancer cell lines and tissues. (**A**) Average insulation score profile of TAD boundaries based on their differential cluster in MCF10 progression and in triple negative breast cancer (TNBC) cell lines and primary patient tissues (Kim et al., 2022). (**B**) Boxplots showing the distribution of insulation scores at MCF10 boundaries of various differential clusters. Filled boxes indicate the insulation scores from TADs in each differential cluster, while empty boxes indicate the distribution of scores at the same number of static TAD boundaries. Stars indicate cell lines where there is a significant difference between the insulation score of boundaries and the static matched set (p ≤ 0.05, Wilcoxon rank sum test). (**C**) Aggregate plots showing the log_2_ of observed/expected counts at MCF10 boundaries in MCF10 cell lines as well as cell lines from Kim et al., 2022.

We identified features of chromatin organization at several levels, including large-scale reorganizations of compartments, medium-scale changes in TADs, and fine-scale changes in chromatin loops (Figure 1B). We assigned A and B compartments using CALDER at a resolution of 10 kb, TADs using SpectralTAD combined with FAN-C boundary insulation score (IS) calculations at 10 kb, and loops using SIP at 5- and 10-kb resolution (see Methods for details). Each cell type had similar percentages of the genome assigned to the active A (47.1–50.2%) or inactive B (49.7–52.9%) compartment, with the two cancer cell types MCF10AT1 and MCF10CA1a having a slightly higher proportion of A compartment designations (Figure 1C). Similarly, we detected a similar number of TADs (between 7459 and 7825) and chromatin loops (between 15,713 and 17,332) in each cell line (Figure 1D, E). Although the three cell lines are karyotypically similar due to their shared genetic background, they contain large-scale chromosomal duplications and translocations which were identified by SKY karyotyping analysis (Figure 1—figure supplement 1B), and numerical chromosome aberrations based on SKY and Micro-C sequencing depth analysis were included in the analysis of all chromatin features (see Methods; Figure 1—figure supplement 1C, Supplementary file 2). After this correction, chromatin loop counts showed a high degree of reproducibility between technical and biological replicates (Figure 1—figure supplement 1D). We used this deep dataset to characterize structural features that are reshaped during cancer progression.

### Cancer progression reorganizes compartments, TADs, and chromatin loops

Comparative analysis across the three cell types identified significant changes in all major chromatin features during cancer progression (Figure 1, Figure 1—figure supplement 2A, B, Supplementary files 3–5).

At a large-scale, we detected changes in compartmentalization. We observed a general shift towards the more active A compartment in early cancer progression, with a larger portion of genomic regions becoming more A-like (31.0%) compared to more B-like (26.0%) in the transition from MCF10A to MCF10AT1, while these changes are more balanced in the later transition from MCF10AT1 to MCF10CA1a (30.0% and 30.6%, respectively). Interactions within the most A- and B-like compartments were predominant in the pre-cancerous MCF10A cells (Figure 1F). However, in MCF10AT1 and MCF10CA1a, stronger interactions appear between more intermediate regions, suggesting a greater degree of intermixing that is consistent with increased compartmental heterogeneity which appears to occur early during cancer progression (Figure 1F; Johnstone et al., 2020; Sen et al., 2024; Liu et al., 2025).

TAD boundaries represent genomic regions where upstream and downstream sequences are partially insulated from one another, with fewer contacts between them than within (Dekker and Mirny, 2016; Rowley and Corces, 2018; da Costa-Nunes and Noordermeer, 2023). We detected a total of 13,231 TADs across all three cell types, with 17,097 unique boundaries. TADs detected range in size from 190 kb to as large as 3.8 Mb, with a mean of 663 kb and a median of 460 kb (Figure 1—figure supplement 2C). Assessing changes in IS at TAD boundaries revealed 3392 (19.8%) boundaries where the degree of insulation changed significantly over the course of cancer progression. Because individual boundaries may be simultaneously used by multiple TADs, the total number of TADs which changed during progression is 5084 (38.5%) (Figure 1G). There are nearly three times as many boundary changes between later stages in cancer progression (1693 differential boundaries between MCF10AT1 and MCF10CA1a) than early stages (567 between MCF10A and MCF10AT1). Interestingly, TAD boundaries that gained or lost insulation during progression showed a significant enrichment for weakened boundaries (71.2%) with far fewer boundaries exhibiting increased insulation strength as cancer progressed (28.8%; p-value 0, permutation test, Figure 1—figure supplement 2D). This late-stage weakening of boundaries may reflect a more heterogeneous cell population as cancer progresses (Corces et al., 2016; Bose et al., 2024; Dagogo-Jack and Shaw, 2018; van den Brand et al., 2025; Choppavarapu et al., 2025).

Chromatin loops are formed by two distal genomic regions that are in more frequent contact than their surrounding or intervening sequences, indicated by higher contact frequency (Dekker and Mirny, 2016; Misteli, 2020; Davidson and Peters, 2021; de Wit and Nora, 2023). We found 29,205 chromatin loops across all three cell lines, ranging in size from 50 kb to 2 Mb, with a mean of 402 kb and median of 270 kb in length (Figure 1—figure supplement 2C). 77.6% of loop anchors coincided with CTCF peaks, representing 95.0% of loops with at least one anchor bound by CTCF, and CTCF-bound loops were stronger and longer than non-CTCF loops (Figure 1—figure supplement 2E, F). Loop boundaries often overlapped with TAD boundaries, with 52.4% of TADs consisting of loop domains across all cell lines. However, a majority (73.0%) of chromatin loops did not include TADs (Figure 1—figure supplement 2G). TADs without loop interactions at their boundaries tended to be larger, while loops without TADs can be found at all sizes but are enriched for shorter loops (Figure 1—figure supplement 2H).

To identify loops that changed significantly during cancer progression, we assessed changes in contact frequency among every loop in each cell type, correcting for karyotypic differences that result in differences in coverage between cell lines (see Methods). We identified 1469 chromatin loops that change significantly over the course of cancer progression, including both weakened and strengthened contacts (Figure 1H), representing 5.0% of all identified loops. Differential loops were defined by a ≥1.5 fold-change between contact frequencies in any two MCF10 cell lines, and an adjusted p-value of <0.025 when considering variation across biological and technical replicates. Unlike TADs, there was a more balanced number of changes between early (1004 differential loops between MCF10A and MCF10AT1) and late (1204 between MCF10AT1 and MCF10CA1a) progression stages, as well as between strengthened (679 loops, 46.2% of all differential loops) and weakened loops (790 loops, 53.8% of all differential loops). Interestingly, only a small portion (19.0%) of differential loops were accompanied by changes in CTCF binding (Figure 1—figure supplement 2I). Motif analysis of differential loop anchors revealed only weak motifs of various transcription factors enriched at the boundaries of gained and lost loops, although occupancy did not appear high enough to explain most of the changes we observe (Figure 1—figure supplement 2J). Weakened loops were often associated with a decrease in H3K27ac, a mark of active enhancers, consistent with the notion that active enhancers can help recruit loop extrusion machinery (Figure 1—figure supplement 2K; see below) (Georgiades et al., 2023; Rinzema et al., 2022).

Taken together, these results demonstrate significant global changes in genome organization during cancer progression across multiple scales from chromatin compartments to loops.

### Comparison to breast cancer patient data

To assess how generalizable the static and dynamic structures detected in the MCF10 progression series are to human tumors, we examined the chromatin loops and TAD boundaries from the MCF10 progression series in non-cancerous mammary epithelial cells (HMEC), five cell lines representing distinct cancer subtypes ranging from less (luminal, HER2+) to more aggressive (triple negative), as well as tissue samples from triple negative breast cancer (TNBC) patients with contralateral normal controls (Figure 1—figure supplements 3 and 4; Kim et al., 2022).

We found that most loops and TAD boundaries detected in MCF10 cells had conserved signatures in each of the other cell types, with chromatin loops generally showing high observed-versus-expected contact frequencies and boundaries showing strong dips in IS (Figure 1—figure supplements 3A and 4A, B). When we compared the profiles of differential MCF10 features relative to static MCF10 features within each cell type, we found some cell-line specific changes. For example, loops in the ‘up-early’ cluster, which are weaker in MCF10A and stronger in both MCF10AT1 and MCF10CA1a, were significantly stronger than static loops among patient TNBC cells (Figure 1—figure supplement 3B; p-value ≤0.05, Wilcoxon rank sum test). This loop cluster was also stronger in HER2+ (HCC1954) and TNBC A (HCC70) cell lines, and the ‘down-early’ loop cluster is significantly weaker than static loops. Among differential TAD boundaries, those that have strong insulation in MCF10A and MCF10AT1 cells, but reduced insulation in MCF10CA1a, showed stronger insulation among all other examined cancer cell types when compared to static loop boundaries, suggesting that the boundaries that weaken in MCF10CA1a are not necessarily consistently weakened in breast cancer cell lines and tissues (Figure 1—figure supplement 4C; p-value ≤0.05, Wilcoxon rank sum test). These findings highlight that different model systems indeed have distinct profiles of structural change, just as they have distinct gene expression profiles.

### Chromatin loops connect gene promoters to distal regulatory features

We then sought to explore how long-range chromatin interactions relate to gene expression. We identified 17,185 expressed genes across any of the three MCF10 cell types using RNA-Seq and 8840 differentially expressed genes across all pairwise comparisons in the MCF10 cancer progression (see Methods; Figure 2—figure supplement 1A, B, Supplementary file 6). A similar number of genes changed in later stages of cancer development (4968 between MCF10AT1 and MCF10CA1a) compared to early progression (4773 between MCF10A and MCF10AT1). Reassuringly, as expected from previous studies (Fritz et al., 2018; Rhee et al., 2008), genes associated with epithelial morphogenesis and cell adhesion were upregulated early during progression, whereas regulation of differentiation, tissue development, metabolism, and signal transduction genes was observed during later stages of progression (Figure 2—figure supplement 1C). These changes are consistent with the development of an intermediate and diverse pre-cancerous state early on during progression, while late changes are known to facilitate metastasis and support the epithelial-to-mesenchymal-like transition observed phenotypically among the progression series (Figure 2—figure supplement 1D; Ferlier and Coulouarn, 2022; Gavish et al., 2023; Liberzon et al., 2015; Ma et al., 2003).

To assess clinical significance of the differentially expressed genes in the MCF10 progression system, we overlapped this gene set with data from the Cancer Genome Atlas breast cancer cohort (TCGA-BRCA) (Ciriello et al., 2015; Koboldt et al., 2012). A total of 1884 genes that are differentially expressed between any stages of the MCF10 progression series were also differentially expressed between normal and tumor samples from breast cancer patients, but only roughly half change in the same direction (see Methods; Figure 2—figure supplement 1E). Interestingly, we found a higher degree of directional agreement between late changing genes (i.e. genes that change in MCF10CA1a compared to MCF10A and MCF10AT1) than early changing genes (i.e. genes that change in MCF10AT1 and MCF10CA1a compared to MCF10A). Of note, several looped genes which are differentially expressed in the MCF10 series showed an effect on overall patient survival (Figure 2—figure supplement 2A–D), and all but one of these genes exhibit significant differences in expression between normal and tumor TCGA-BRCA samples (Figure 1—figure supplement 2E; two-sampled Wilcoxon test of normalized counts, see Methods).

**Figure 2.**
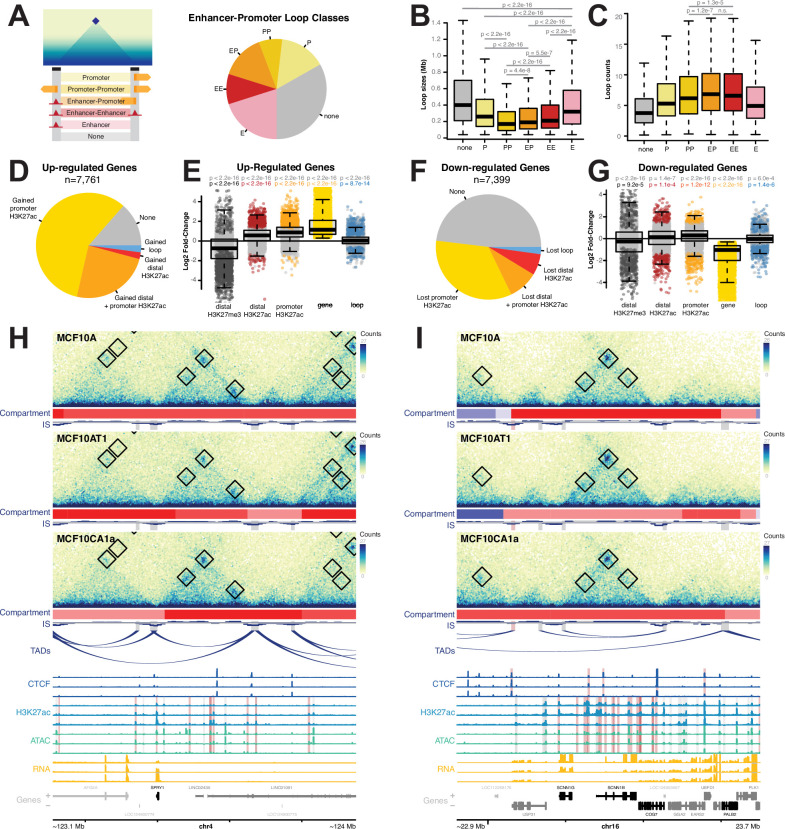
Persistent chromatin loops connect differentially expressed genes to distal enhancers. (**A**) Percentages of loops designated as either promoter–promoter, enhancer–promoter, enhancer–enhancer, or single-sided promoter or enhancer loops. (**B**) Distributions of loop sizes by enhancer/promoter designations. p-values represent Wilcoxon tests comparing the means of different loop classes. Boxplots show the median (middle line), 25th and 75th quartiles (box perimeters), and range excluding outliers (dashed line whiskers). Outliers are defined as values that are over 1.5 times the interquartile range beyond the box bounds and are excluded from these plots. p-values represent Wilcoxon tests comparing the means of various loop sets. Non-significant (n.s.) represents p-values above 0.05. (**C**) Distributions of loop strength by enhancer/promoter designations. p-values represent Wilcoxon tests comparing the means of various loop sets. Non-significant (n.s.) represents p-values above 0.05. (**D**) Percentages of upregulated genes that have gained H3K27ac at promoters, distal enhancers, both, or gained loops. (**E**) Log_2_ fold-change of distal H3K27me3 (gray), distal H3K27ac (red), promoter H3K27ac (orange), gene expression (yellow), and loop strength (blue), when overlapped. Gray dots indicate features that do not change significantly, while colored points are significantly differential features. Boxplots are defined as in (**B**). p-values represent Wilcoxon tests comparing the means of each class to 0. Non-significant (n.s.) represents p-values above 0.01. (**F**) Percentages of downregulated genes that have gained H3K27ac at promoters, distal enhancers, both, or gained loops. (**G**) Log_2_ fold-change of distal H3K27me3 (gray), distal H3K27ac (red), promoter H3K27ac (orange), gene expression (yellow), and loop strength (blue), when overlapped. Boxplot details as defined in (**E**). (**H**) An example of an upregulated gene (SPRY1) connected to gained enhancers by static loops. Black boxes show loop annotations. Red compartment tracks indicate A compartments, while blue indicates B compartments. In CTCF signal tracks, red highlights indicate differential CTCF peaks. In H3K27ac and ATAC-Seq signal tracks, red highlights indicate differential enhancers as determined by changes in H3K27ac. Genes highlighted in black are differentially expressed. (**I**) An example of downregulated genes (SCNN1G and SCNN1B) connected to lost enhancers by static loops. Plot annotations are as described in (**H**).

To explore the functional role of chromatin loops, we next overlapped chromatin loops with expressed and differential gene promoters and potential regulatory regions. We identified 52,953 potential enhancers as defined by overlapping histone H3K27ac ChIP-Seq and ATAC-Seq accessibility peaks, commonly used to identify enhancer regions (see Methods) (Creyghton et al., 2010; Glossary, 2025; Heintzman et al., 2007). Approximately 66.8% of chromatin loops featured some combination of active gene promoters and enhancers within 10 kb of loop anchors (Figure 2A). We found that chromatin loops that connect two features (either enhancers or promoters; mean length 260–311 kb) are typically shorter than those that contain only one feature (mean length 376–435 kb) or none (Figure 2B; mean length 532 kb; p-value for all comparisons <2.2e−16, Wilcoxon rank sum test), with promoter–promoter loops being the smallest on average (mean length 260 kb). Interestingly, enhancer–promoter and enhancer–enhancer loops (mean counts 7.9 and 7.9, respectively) were stronger than promoter–promoter loops (mean counts 7.2) despite being longer on average, suggesting that epigenetic signatures associated with active enhancers may support stronger contacts (Figure 2C; p-value for both comparisons <1.3e−5, Wilcoxon rank sum test).

**Figure 2—figure supplement 1.**
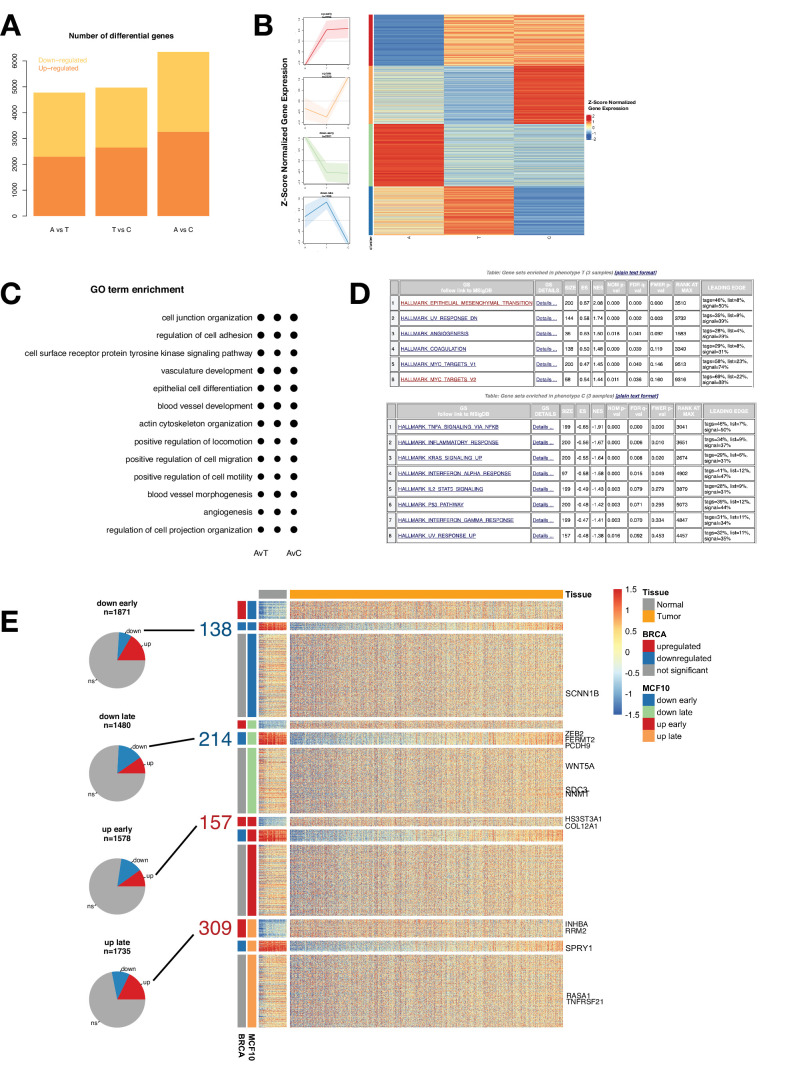
Differential gene expression patterns across breast cancer progression include cancer-relevant pathways. (**A**) The number of genes that exhibit a significant increase (orange) or decrease (gold) in expression between each pairwise comparison of cell types. (**B**) Differential genes clustered by their timing of change, depicted in line plots and heatmap. (**C**) Gene ontology (GO) term enrichment for genes differentially expressed in each pairwise comparison of cells. (**D**) Gene set enrichment analysis (GSEA) results showing gene sets enriched among genes differentially expressed early (MCF10AT1, top) or late (MCF10CA1a, bottom) in breast cancer progression. (**E**) A heatmap shows the expression of all differential genes in the MCF10 progression series among normal (gray) and tumor (gold) tissue samples from the Cancer Genome Atlas breast cancer cohort. Genes are clustered first by their pattern of change in MCF10, then their differential status in the patient data. Example genes used in the study are listed on the right. Each cluster also shows a pie chart on the left depicting the percent of genes that are shared in the patient data, with genes that change in the same direction highlighted.

**Figure 2—figure supplement 2.**
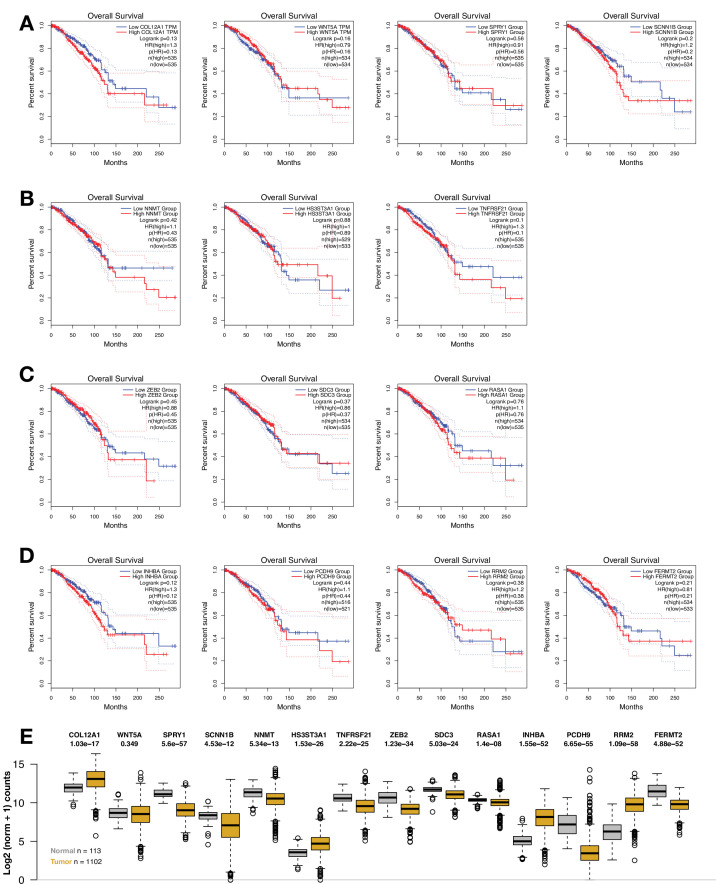
Clinical relevance of select genes misregulated in MCF10. Overall survival curves from the Cancer Genome Atlas for fourteen select genes highlighted in the main figures (**A**), Figure 4—figure supplement 2 (**B, C**), and Figure 2—figure supplement 3 (**D**). (**E**) Gene expression data from the Cancer Genome Atlas for fourteen select genes. Gray boxplots represent expression values from normal samples and gold represent tumor samples. p-values represent the Wilcoxon rank-sum test comparing the normal and tumor expression levels for each gene.

**Figure 2—figure supplement 3.**
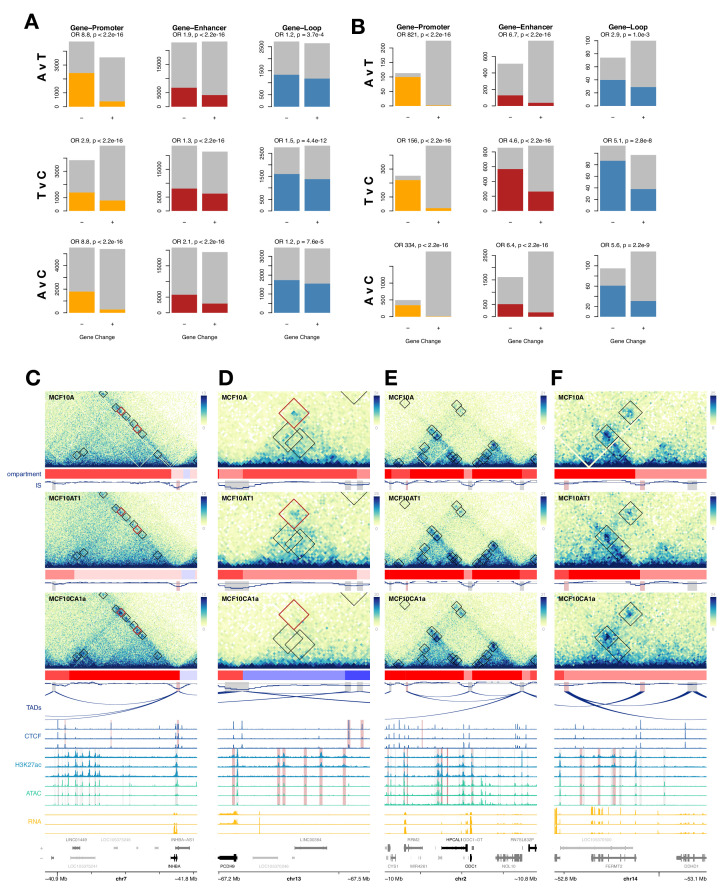
Differentially expressed genes overlap differential and static loops with differential distal regulatory regions. (**A**) The number of promoter H3K27ac peaks (left), distal enhancer H3K27ac peaks (middle), or loops (right) that change in a positive (gray) or negative (gold, red, blue) direction based on their overlap with up- or downregulated genes. (**B**) Same plots as (**E**) but subset for significantly differential features. (**C–F**) Examples of genes that are differentially regulated in the same direction in MCF10 progression and patient samples and which overlap with chromatin loops in MCF10 cell lines. Black boxes show loop annotations, while red boxes indicate differential loops. Red compartment tracks indicate A compartments, while blue indicates B compartments. In CTCF signal tracks, red highlights indicate differential CTCF peaks. In H3K27ac and ATAC-Seq signal tracks, red highlights indicate differential enhancers as determined by changes in H3K27ac. Genes highlighted in black are differentially expressed.

**Figure 2—figure supplement 4.**
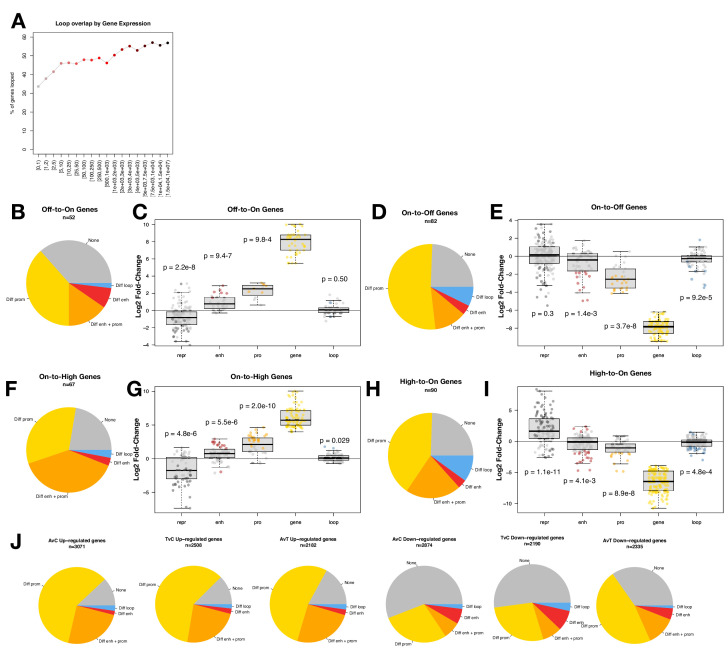
Relationship between differential gene expression, chromatin loops, and distal enhancers is consistent based on expression levels and cancer stages. (**A**) Percentage of genes that overlap with loop anchors based on gene expression level. (**B**) Percentages of off-to-on genes that have gained H3K27ac at promoters, distal enhancers, both, or gained loops. (**C**) Log_2_ fold-change of distal H3K27me3 (gray), distal H3K27ac (red), promoter H3K27ac (orange), gene expression (yellow), and loop strength (blue), when overlapped. Gray dots indicate features that do not change significantly, while colored points are significantly differential features. p-values represent Wilcoxon tests comparing the means of each class to 0. Non-significant (n.s.) represents p-values above 0.01. (**D–I**) Same as (**B, C**), but for on-to-off genes (**D, E**), on-to-high genes (**F, G**), and high-to-on genes (**H, I**). (**J**) Percentages of upregulated genes (left) and downregulated genes (right) that have correlated changes in H3K27ac at promoters (gold), distal enhancers (red), both (orange), or loop contact frequency (blue), by pairwise comparison of cell types.

**Figure 2—figure supplement 5.**
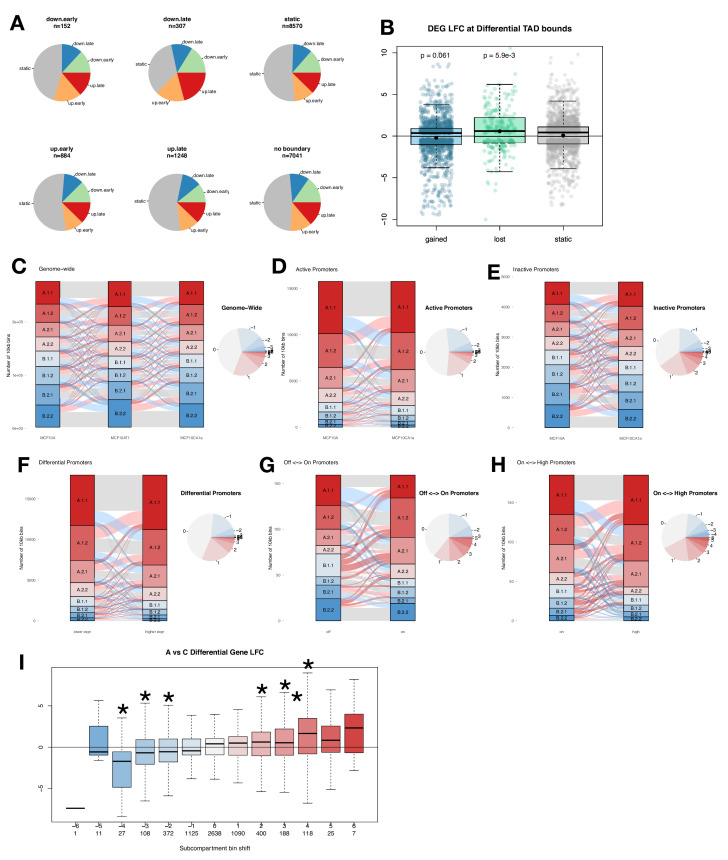
Differential topologically associating domain (TAD) boundaries and subcompartment shifts have a subtle relationship to gene expression. (**A**) Pie charts showing the differential status of genes within 50 kb of TAD boundaries of various differential clusters. (**B**) Boxplots of the log_2_ fold-change of genes at the boundaries of TAD boundaries that are either weakened (blue), strengthened (green), or static (gray). p-values represent Wilcoxon rank sum tests comparing the mean of each set to the static set. (**C**) Alluvial plots showing the transition of subcompartments from MCF10A to MCF10AT1 and from MCF10AT1 to MCF10CA1a. Gray ribbons indicate bins that have the same subcompartments between cell types, while red ribbons shift more A-like and blue ribbons shift more B-like. Darker ribbons shift by more than one subcompartment. A pie chart to the right summarizes how many subcompartments all bins shift by, with negative numbers (blue) indicating shifts towards more B-like compartments and positive numbers (red) indicating shifts towards more A-like compartments. Alluvial plot showing the difference in subcompartments for the promoters of (**D**) actively expressed or (**E**) non-expressed genes that have similar gene expression in MCF10A and MCF10CA1a. Plots and pie charts are colored as in (**C**). Alluvial plots showing the difference in subcompartments for the promoters of genes that are differentially expressed between any two cell types (**F**), change from on to off (**G**), or change from on to high (**H**). For each figure, the left bar shows the subcompartments in the cell line with lower expression and the right bar is from the cell line with higher expression. The pie charts summarize the shifts as in (**C**). (**I**) A boxplot indicating the distribution of gene log_2_ fold-change between MCF10A and MCF10CA1a for genes with promoters in bins that shift by various numbers of subcompartments. Negative numbers (blue) indicate bins that shift to more B-like subcompartments, while positive numbers (red) indicate shifts to more A-like subcompartments. The numbers below indicate the number of bins that fit into each category. Stars indicate significant differences in mean gene log_2_ fold-change compared to genes that do not change subcompartments between the two cell types (gray), as indicated by a p-value of 0.01 or less (Wilcoxon rank sum test).

### Differentially expressed genes show proximal and distal epigenetic changes at persistent chromatin loops

To understand the regulatory modes of action of genes which were differentially expressed during cancer progression, we determined if they had gained or lost the activity-associated H3K27ac mark at their promoters or at distally looped enhancers. We found that while many genes only featured a corresponding change in H3K27ac at their promoter (57.9% of upregulated and 34.1% of downregulated genes), a large percentage also showed changes in distal enhancer activity (26.5% of upregulated and 15.7% of downregulated genes), suggesting that enhancer loops may be playing an important functional role in control of these genes (Figure 2D, F). Comparing the direction of fold-change for genes and promoter H3K27ac, distal H3K27ac, or contact frequency with distal enhancers using Fisher’s Exact test revealed odds ratios significantly higher than 1 for all comparisons (8.8, 2.1, and 1.2, respectively, for changes between MCF10A and MCF10CA1a), but that there was a stronger association with promoters than enhancers or loop strength (Figure 2—figure supplement 3A, B). This trend was similar for genes that were differentially regulated both early and late, suggesting that the role of chromatin loops is consistent across all stages of cancer progression. In support of the finding that both distal regulatory changes and changes in contact frequency appear to contribute to changes in gene expression, looped genes that are regulated similarly in MCF10 progression and patient data include both up- and downregulated genes anchored by both static (i.e. *RRM2* and *FERMT2*) and dynamic chromatin loops (i.e. *INHBA* and *PCDH9*; Figure 2—figure supplement 3C–F).

To further assess the contribution of looped regulatory regions, we explored the degree of change in epigenetic marks and contact frequency between proximal and distal regions. Comparing the changes in acetylation at all gene promoters and distal regulatory regions revealed that upregulated genes exhibit a significant increase in H3K27ac at distally looped enhancers, as well as a significant loss of repressive H3K27me3 marks (Figure 2E; p-value <2.2e−16, one-sample Wilcoxon test). This trend is weaker for downregulated genes, which feature a closer balance of gained and lost H3K27ac at both enhancers and promoters, as well as both gained and lost H3K27me3 at distal regions (Figure 2G). These results suggest that the static chromatin structures observed during the cancer progression process contribute to the control of differentially regulated genes, particularly among upregulated genes.

We observed that genes that are more strongly expressed are more likely to overlap with the anchor of a chromatin loop, ranging from roughly 33.4% overlap for genes with 0 read counts, 47.0% for genes with 10–1000 counts, and 56.2% for genes with 5000 and more counts (Figure 2—figure supplement 4A). To explore whether differential loops are more prominent among genes that change from a low active state to higher expression bins, we analyzed 108 genes that went from an unexpressed or ‘off’ state (2 or fewer read counts) in one cell line to an expressed ‘on’ state (100 or more read counts) in another (Figure 2—figure supplement 4B–E). While these genes were not enriched for differential loops, over 40% overlap with static loops. Similarly, genes that change from a modest ‘on’ state to high expression levels (1000 or more read counts) are not enriched for differential loops; however, they do exhibit a higher static loop overlap (61.8%) consistent with higher total gene expression levels (Figure 2—figure supplement 4F–I). For all gene sets examined, looped genes showed strong and similar trends at distal regulatory regions.

Given that only 5% of loops changed significantly during progression (see Figure 1), it is not surprising that only a small percentage of differentially expressed genes exhibited significant changes in chromatin contacts with distal enhancers (2.1% of upregulated and 2.2% of downregulated genes; Figure 2D, F). This trend was similar between both early- and late-regulated genes (Figure 2—figure supplement 4J). On average, there is a slight but significant change in contact frequency between gene promoters and distal enhancers that corresponds to the change in gene expression (Figure 2E, F). However, most differentially expressed genes are in regions where chromatin structure is essentially stable, reinforcing that persistent structural changes are not universally required for gene regulation.

For example, the *SPRY1* gene, which regulates cell growth and differentiation and has been shown to be upregulated in triple-negative breast cancer tumors (He et al., 2016), is upregulated between MCF10AT1 and MCF10CA1a, and is statically looped to distal enhancers that gain H3K27ac (Figure 2H). Similarly, the *SCNN1G* gene, which encodes for a subunit of a sodium channel and is suppressed in head and neck squamous cell cancer (Yang et al., 2022), is downregulated between MCF10AT1 and MCF10CA1a, and is statically looped to distal enhancers that lose H3K27ac (Figure 2I). In both cases, the contact frequency remains relatively constant despite changes in distal enhancer acetylation. Many additional examples of statically looped, differentially expressed genes were found, including *RRM2* and *FERMT2* (Figure 2—figure supplement 3E, F). Taken together, our results show that changes in gene expression are not necessarily accompanied by structural changes, and they suggest that stable chromatin loops may facilitate functionally relevant gene expression programs by providing a pre-existing structure through which differentially regulated distal enhancers can act on target genes.

### Changes in TAD boundary insulation and subcompartments have subtle impacts on gene expression

To explore the impact of changes in insulation at domain boundaries on gene expression, we next examined genes within 50 kb of differential boundaries. We found that genes close to weakened boundaries were not enriched for differentially expressed genes, but those near strengthened boundaries were (Figure 2—figure supplement 5A; p-values of 0.141 for early and 0 for late strengthened boundaries, and p-values of 1 for weakened boundaries, permutation test). While strengthened boundaries featured both upregulated and downregulated genes, there was a small but significant correlation between the strength of changing boundaries and fold-change of expression of nearby genes when compared to genes at static boundaries, suggesting an average positive impact on gene expression (Figure 2—figure supplement 5B; p-value 5.9e−3, Wilcoxon rank sum test). This suggests that changes in TAD boundary insulation have small but noticeable impacts on gene expression.

We also examined how subcompartments change genome-wide and at gene promoters. We see that between any two cell types, a majority of subcompartment changes are small changes, for example from A.2.2 to A.2.1 (1 step more A-like) or B.1.1 (1 step more B-like), with larger shifts being rarer (Figure 2—figure supplement 5C). The promoters of active genes are enriched for A.1.1 and A.1.2 subcompartments but depleted for all B subcompartments, while inactive gene promoters closely resemble genome-wide distributions (Figure 2—figure supplement 5D, E). The promoters of differentially expressed genes have similar subcompartments at lower and higher expression levels, but these changes are more drastic for genes that shift from on to off or on to high, as defined above (Figure 2—figure supplement 5F–H). Differentially expressed genes with promoters that shift to more B-like by 2 or more subcompartments or more A-like by 3 or more subcompartments have significant impacts on fold-change, but smaller shifts have minimal impacts on gene expression (Figure 2—figure supplement 5I; p-value ≤0.01, Wilcoxon rank sum test). In summary, small changes in subcompartments are very common but appear to have little impact on gene expression, while larger changes correlate more strongly with changes in gene expression during breast cancer progression but are comparatively rare.

### Changes in enhancer acetylation and enhancer–promoter contact are associated with changes in gene expression

To begin to distinguish the effects of enhancer–promoter contact and chromatin looping from enhancer activity effects, we compared gene expression changes at looped and non-looped enhancer–promoter pairs. To do so, we used the activity-by-contact (ABC) model to predict functional enhancer–promoter pairs. ABC combines estimates of enhancer accessibility and activity from ATAC-Seq and H3K27ac ChIP-Seq with enhancer–promoter contact frequency from Micro-C data to generate an estimate of the likelihood of functional enhancer–gene interactions (see Methods) (Fulco et al., 2019). This method allowed us to identify distal regulatory regions that are functionally linked to gene promoters without specifically requiring overlap with chromatin loops. For example, an enhancer and promoter may be in high contact as measured by Micro-C because they overlap with loop anchors, or because they are at close genomic distance. For this analysis, potential enhancers were defined as any overlapping H3K27ac ChIP-Seq and ATAC-Seq peaks 750 bp or farther from the transcription start sites of the target gene.

Applying the ABC model to our data identified 150,056 potential enhancer–promoter pairs across all three cell types. Of these, 53.4% are also promoters of other genes, 23.7% are within the bodies of other genes, and 22.9% are intergenic, and range in distance from 750 to 5 M base pairs away from target gene promoters (Figure 3—figure supplement 1A). To better understand the relationship between contact frequency, enhancer activity, and gene expression, we asked how changes in enhancer activity or contact relate to gene expression at target promoters.

**Figure 3.**
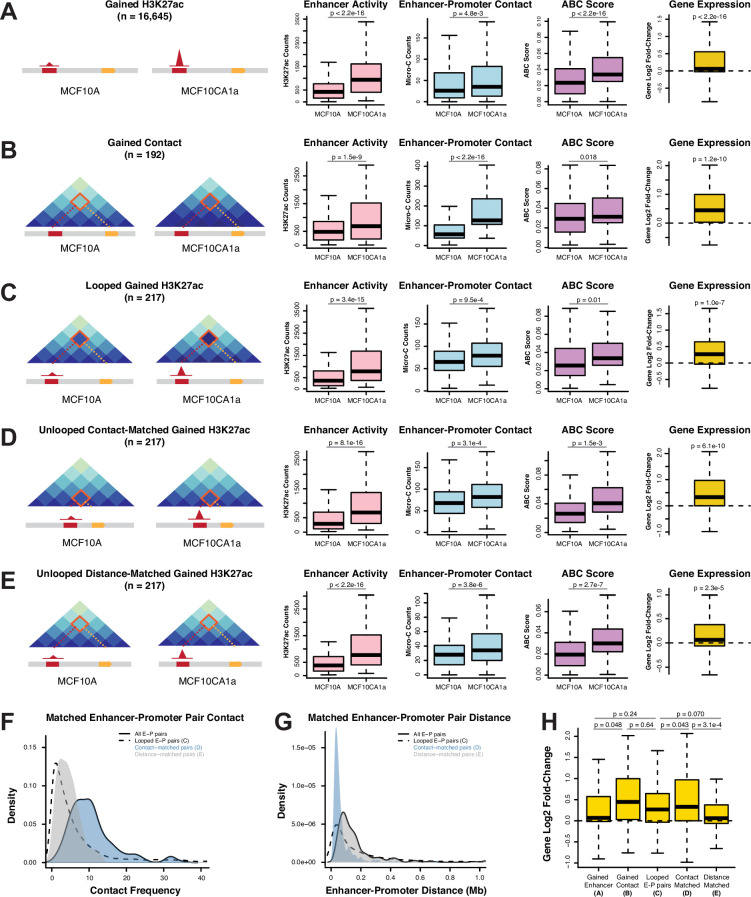
Changes in enhancer acetylation or enhancer–promoter contact are associated with changes in gene expression. Boxplots of distal enhancer H3K27ac (pink), enhancer–promoter contact (blue), and activity-by-contact (ABC) score (purple), as well as gene log_2_ fold-change (yellow) for enhancer promoter pairs that feature (**A**) differential H3K27ac among enhancers, (**B**) differential enhancer–promoter contact frequency, and (**C**) differential H3K27ac for enhancer–promoter pairs supported by a chromatin loop. Boxplots in (**D**) and (**E**) represent sets of non-looped enhancer–promoter pairs with differential H3K27ac that are matched to the looped set in (**C**) by contact and distance, respectively. Boxplots show the median (middle line), 25th and 75th quartiles (box perimeters), and range excluding outliers (dashed line whiskers). Outliers are defined as values that are over 1.5 times the interquartile range beyond the box bounds and are excluded from these plots. p-values represent *T*-tests comparing the means of values in MCF10A and MCF10CA1a for enhancer activity, enhancer–promoter contact, and ABC score, and *T*-tests comparing the mean of the value to 0 for gene log_2_ fold-change. (**F**) Contact distribution of all enhancer–promoter pairs (dashed line), compared to the looped enhancer–promoter pairs in (C, solid line), the contact-matched pairs in (D, blue shade), and the distance-matched pairs in (E, gray shade). (**F**) Distance distribution of all enhancer–promoter pairs (dashed line), compared to the looped enhancer–promoter pairs in (C, solid line), the contact-matched pairs in (D, blue shade), and the distance-matched pairs in (E, gray shade). (**G**) Summary boxplot of the gene log_2_ fold-change for the enhancer–promoter pairs previously shown in figures (**A–E**). p-values represent *T*-tests comparing the means of average gene log_2_ fold-changes values for different sets of enhancer–promoter pairs.

Pairwise comparison of the MCF10 progression lines indicated that changes in both contact frequency and enhancer activity appear to drive changes in enhancer–promoter networks predicted by ABC (Figure 3). For example, observing potential enhancers with changes in H3K27ac between MCF10CA1a and MCF10A reveals that these enhancers also exhibit a change in contact frequency and are associated with upregulation of target genes (Figure 3A). We also found that changes in contact frequency are associated with increases in H3K27ac and correlate with higher gene expression (Figure 3B). These results show that not only changes in either contact frequency and enhancer activity correlate with increased gene expression, but they also correlate with each other, suggesting a potentially linked functional role during enhancer–promoter communication.

**Figure 3—figure supplement 1.**
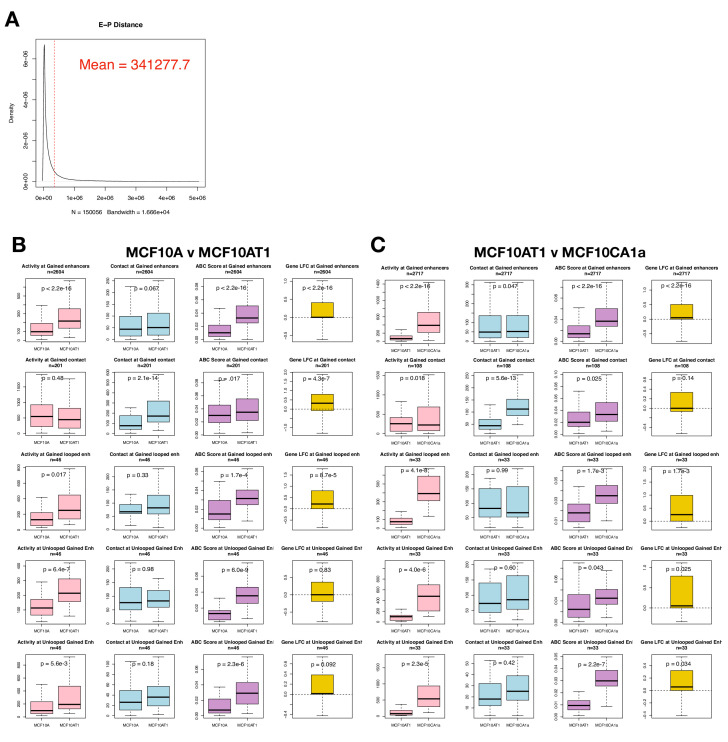
Enhancer–promoter pair details. (**A**) Distribution of enhancer–promoter distances as predicted by the activity-by-contact (ABC) model. (**B**) Boxplots of distal enhancer H3K27ac (pink), enhancer–promoter contact (blue), and ABC score (purple), as well as gene log_2_ fold-change (yellow) for enhancer promoter pairs that feature (top to bottom) differential H3K27ac among enhancers, differential enhancer–promoter contact frequency, differential H3K27ac for looped enhancer–promoter pairs, contact-matched non-looped enhancer–promoter pairs, and distance-matched non-looped enhancer–promoter pairs. Comparison shows changes between MCF10A and MCF10AT1. (**C**) Same as (**B**) but showing comparisons between MCF10AT1 and MCF10Ca1a.

To then relate enhancer–promoter pairs to chromatin loops and to orthogonally assess whether chromatin loops are acting as a functional bridge for active enhancers, we compared looped and non-looped enhancer–promoter pairs. Enhancer–promoter pairs that have changes in distal H3K27ac and are supported by chromatin loops correlated with changes in gene regulation (Figure 3C). This effect was stronger than distance-matched non-looped enhancer–promoter pairs, but similar to contact-matched non-looped pairs, suggesting that increased contact frequency caused by loop extrusion may contribute to the stronger correlation with gene expression (Figure 3D, E). Contact-matched non-looped pairs were closer on average to the looped pairs of similar contact frequency, while distance-matched non-looped pairs were in less-frequent contact than looped pairs of similar genomic distance (Figure 3F, G).

Comparing the distributions of target gene fold-change for these various sets of enhancer–promoter pairs revealed several trends (Figure 3H). First, pairs with significant changes in contact have a larger mean gene fold-change than pairs with significant changes in activity, suggesting that either can contribute to changes in enhancer–promoter functional pairing but that contact may have a particularly strong impact. Second, looped enhancer–promoter pairs have a comparable or larger mean gene fold-change to pairs with changes in activity or contact, suggesting again that chromatin loops may support functional enhancer–promoter pairs. Lastly, looped pairs have a similar mean gene fold-change as contact-matched pairs, which in turn have a higher mean gene fold-change than distance-matched pairs, suggesting that the increased contact frequency that chromatin loops provide to enhancer–promoter pairs may be a driving force for the functional connection. These trends hold true for all tested pairwise comparisons between cell types (Figure 3—figure supplement 1B, C).

Taken together, these findings demonstrate that not all gene regulatory changes are accompanied by chromatin reorganization, but when it occurs, reorganization appears to facilitate functional changes.

### Changes in chromatin looping are enriched for progression-associated differentially expressed genes

We next explored how changes in chromatin looping may functionally contribute to gene regulation during cancer progression. To do so, we compared changes in chromatin loop contacts to alterations in expression of progression-associated genes. Overall, while only a small subset of gene promoters overlaps with the anchors of differential chromatin loops (507 genes, 3.0% of expressed genes), those that do are significantly enriched for genes that are differentially expressed during cancer progression based on permutation analysis (331 differentially expressed genes, 65.3% of all differentially looped genes; Figure 4—figure supplement 1A).

**Figure 4.**
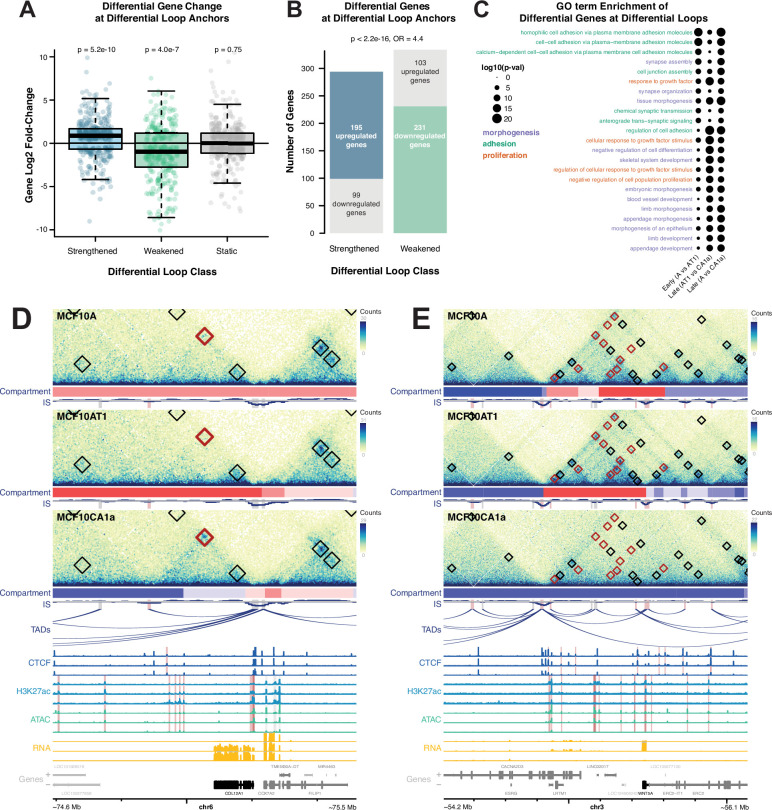
Differential loops are enriched for cancer-relevant differentially expressed genes. (**A**) Log_2_ fold-change of differentially expressed genes at the anchors of gained (blue), weakened (green), or static (gray) loops. Boxplots show the median (middle line), 25th and 75th quartiles (box perimeters), and range excluding outliers (dashed line whiskers). Outliers are defined as values that are over 1.5 times the interquartile range beyond the box bounds and are excluded from these plots. p-values represent Wilcoxon tests comparing the mean of each set to 0. (**B**) Bar plot showing the number of differentially expressed genes at strengthened or weakened loop anchors. Bar segments are colored by whether the gene is changing the same (blue for upregulated in strengthened loops, green for downregulated in weakened loops) or opposite (gray) direction as the loop. p-value represents a Fisher’s exact test for whether the odds ratio (OR) is greater than 1. (**C**) Gene ontology (GO) term enrichments for genes upregulated in MCF10A, MCF10AT1, or MCF10CA1a. Size indicates p-value. Terms are color-coded based on gene type; morphogenesis (purple), proliferation (orange), and cell adhesion (teal). (**D**) An example of an upregulated gene (COL12A1) with a promoter that overlaps a strengthened loop with distal enhancers. Black boxes show loop annotations, while red boxes indicate differential loops. Red compartment tracks indicate A compartments, while blue indicates B compartments. In CTCF signal tracks, red highlights indicate differential CTCF peaks. In H3K27ac and ATAC-Seq signal tracks, red highlights indicate differential enhancers as determined by changes in H3K27ac. Genes highlighted in black are differentially expressed. (**E**) An example of a downregulated gene (WNT5A) with a promoter that overlaps with several weakened loops containing distal enhancers that lose H3K27ac. Plots are annotated as in (**A**).

We asked whether there was a relationship between the formation or loss of loops and differential expression of these loop-associated genes. We indeed found that differential loops are more likely to change in the same direction as the gene (i.e. increased contact frequency with distal regions associated with increased gene expression) (Figure 4A, B). The fold-change of differentially expressed genes which also showed differential looping were significantly higher than a random sampling of differentially expressed genes (Figure 4A). For example, of 3261 genes that were differentially upregulated between MCF10A and MCF10CA1a, loops were significantly strengthened at 98 of these genes and significantly weakened at 31. Similarly, of 3088 downregulated genes, 65 genes overlap weakened loop anchors and 41 genes overlap strengthened loop anchors. In contrast, the number of expected genes at strengthened or weakened loops for a random sampling of genes this size is 38 and 32, respectively (Figure 4—figure supplement 1B, C). We also found a subset of chromatin loops and genes changed in opposite directions (Figure 4B). The genes whose changes in expression correlate with changes in looping are enriched for several cancer-relevant pathways, such as morphogenesis, differentiation, and proliferation (Figure 4C; Hanahan and Weinberg, 2011).

**Figure 4—figure supplement 1.**
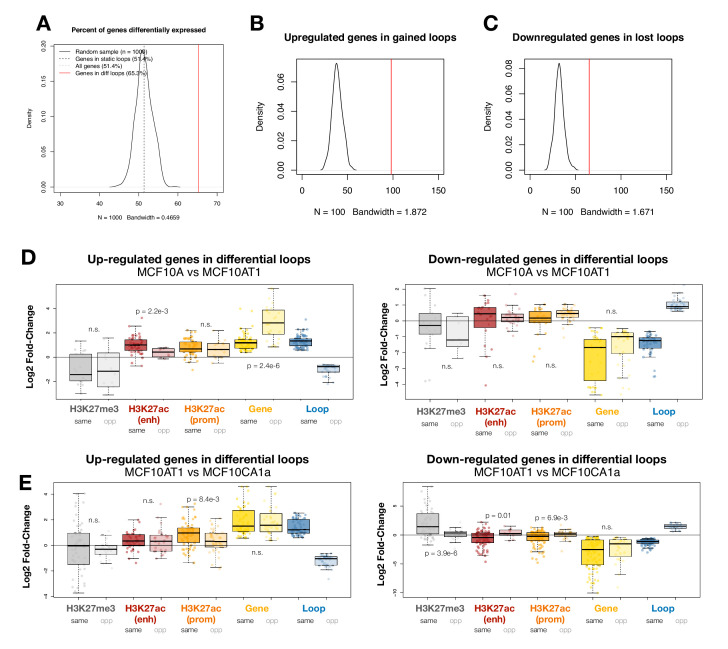
Differential genes within differential loops. (**A**) The number of differentially expressed genes among all genes (dashed light gray line), genes overlapping static loop anchors (dashed dark gray line), genes overlapping differential loop anchors (red line), and a permutation test of a random sampling of a similar number of genes (black line). (**B**) Permutation test results (*n* = 1000) for the number of upregulated genes that overlap with strengthened/gained loops (red) compared to a random sampling (black). (**C**) Permutation test results (*n* = 1000) for the number of downregulated genes that overlap with weakened/lost loops (red) compared to a random sampling (black). Boxplots in (**D**) and (**E**) represent log_2_ fold-change between MCF10A and MCF10AT1 (**D**) or MCF10AT1 and MCF10CA1a (**E**) of distal H3K27me3 (gray), distal H3K27ac (red), promoter H3K27ac (orange), gene expression (yellow), and loops (blue) among upregulated or downregulated genes that overlap with gained loops (darker colors) or lost loops (lighter colors). Boxplots are defined as in (**A**). p-values represent *T*-tests comparing the mean values of the features at loops that change in the same and those that change in opposite directions from the differential genes at their anchors. Non-significant (n.s.) p-values are any p-values above 0.01.

**Figure 4—figure supplement 2.**
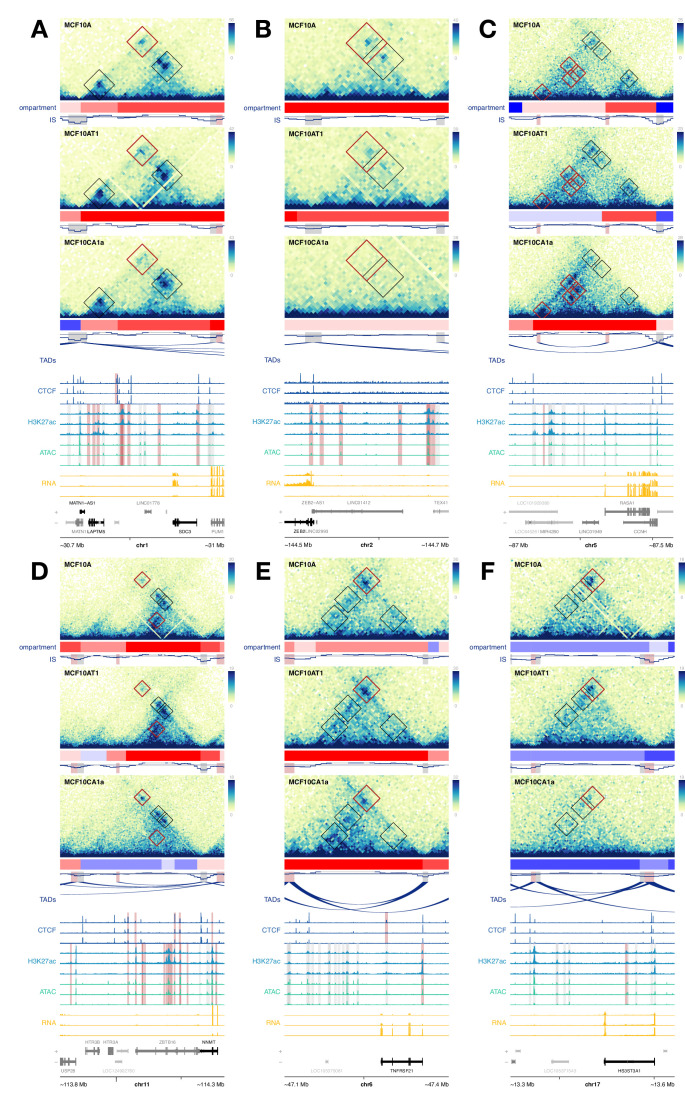
Examples of differentially expressed genes at differential loop anchors. (**A–C**) Examples of genes that are differentially expressed in MCF10 progression and overlap with anchors of differential genes that change in the same direction. Black boxes show loop annotations, while red boxes indicate differential loops. Red compartment tracks indicate A compartments, while blue indicates B compartments. In CTCF signal tracks, red highlights indicate differential CTCF peaks. In H3K27ac and ATAC-Seq signal tracks, red highlights indicate differential enhancers as determined by changes in H3K27ac. Genes highlighted in black are differentially expressed. (**D–F**) Examples of genes that are differentially regulated in MCF10 progression and overlap with anchors of differential genes that change in the opposite direction. Plots are annotated as in (**A–C**).

In total, we identified 127 unique genes upregulated in areas that experience increased chromatin contacts, either at loop anchors or within existing structures. As an example, the promoter of the *COL12A1* gene, which encodes a collagen protein known to be associated with aggressive and mesenchymal phenotypes, overlaps a loop boundary that is very weak in MCF10A cells where *COL12A1* is not expressed (Hu et al., 2022; Papanicolaou et al., 2022; Song et al., 2023). As *COL12A1* gene expression is upregulated during progression, contacts increase at a distal region 310 kb away, and H3K27ac and accessibility also increase at these likely distal regulatory regions (Figure 4D).

Similarly, we observe 123 unique genes that are downregulated during oncogenic progression. One example is *WNT5A,* which encodes for an important signaling protein and is downregulated in breast cancer and across MCF10A progression (Borcherding et al., 2015; Roarty et al., 2009; Zeng et al., 2016). Similar to *COL12A1*, the promoter of *WNT5A* is involved in many differential distal regulatory contacts, ranging in distance from 240 to 640 kb (Figure 4E). Unlike *COL12A1*, these contacts are strongest in MCF10A cells and severely weaken in MCF10AT1 and MCF10CA1a cells. Accompanying these changes in contact, the distal regulatory regions that appear to support *WNT5A* in MCF10A cells become deacetylated and decrease in accessibility. Another example of an upregulated gene that is looped to a distal enhancer is *RASA1*, which gains contact frequency as gene expression increases, while *ZEB2* and *SDC3* are downregulated genes that lose enhancer contact frequency similar to *WNT5A* (Figure 4—figure supplement 2A–C). Other loci showed the opposite behavior; for example, *TNFRSF21* and *HS3ST3A1* are upregulated genes at the anchors of weakened loops, while *NNMT* is a downregulated gene at a weakened loop anchor (Figure 4—figure supplement 2D–F).

### Genome reorganization occurs at cancer-relevant genes and is accompanied by proximal and distal changes in histone marks

Finally, we aimed to comprehensively explore the genes that are differentially regulated in areas that also have strong changes in chromatin looping, to better understand the potential regulatory mechanisms at play.

**Figure 5.**
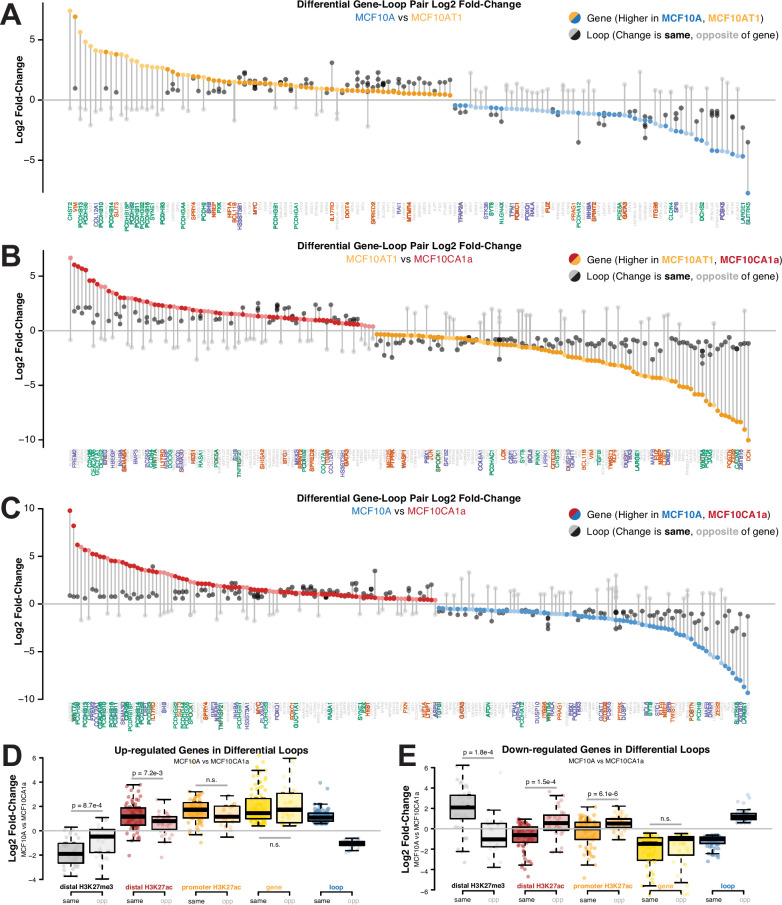
Progression-associated differentially expressed genes exhibit local and distal epigenetic changes at differential loops. Log_2_ fold-change of genes (colored dots) and the differential loops they overlap with (black/gray dots) for genes and loops that change between (**A**) MCF10A and MCF10AT1, (**B**) MCF10AT1 and MCF10CA1a, and (**C**) MCF10A and MCF10CA1a. Gene labels are below. (**D**) Log_2_ fold-change between MCF10A and MCF10CA1a of distal H3K27me3 (gray), distal H3K27ac (red), promoter H3K27ac (orange), gene expression (yellow), and loops (blue) among upregulated genes that overlap with gained loops (darker colors) or lost loops (lighter colors). Boxplots are defined as in (**A**). p-values represent *T*-tests comparing the mean values of the features at loops that change in the same and those that change in opposite directions from the differential genes at their anchors. Non-significant (n.s.) p-values are any p-values above 0.01. (**E**) Log_2_ fold-change of distal H3K27me3 (gray), distal H3K27ac (red), promoter H3K27ac (orange), gene expression (yellow), and loops (blue) among downregulated genes that overlap with gained loops (darker colors) or lost loops (lighter colors). p-values represent *T*-tests comparing the mean values of the features at loops that change in the same and those that change in opposite directions from the differential genes at their anchors. Non-significant (n.s.) p-values are any p-values above 0.01.

A locus-by-locus view of gene and loop fold-change allows us to view the relationship between changes in expression and structure among each pairwise comparison of cells (Figure 5A–C). While we see that a majority of genes have a corresponding change in looping (i.e. upregulated genes overlapping strengthened loops), we observed that the percentage of corresponding changes increases in the later stages of cancer progression. For example, the percentage of differential loop-gene pairs where the gene overlaps at least one gained loop is 47.5% and 69.9% among genes upregulated in MCF10A compared to MCF10AT1 and MCF10CA1a, respectively, 66.7% and 79.6% among genes upregulated in MCF10AT1 compared to MCF10A and MCF10AT1, respectively, and 70.7% and 58.0% among genes upregulated in MCF10CA1a compared to MCF10A and MCF10AT1, respectively. This may indicate that the regulatory impacts of changes in chromatin looping occur over longer timescales, or that genes impacted by changes in chromatin structure may be more common in metastatic cells. We did not, however, find any correlation between the magnitude of loop fold-change and gene fold-change.

To better understand how changes in looping are related to gene expression, we compared patterns of gene expression, promoter H3K27 acetylation, and distal enhancer H3K27 acetylation or trimethylation at looped genes that change in either the same or opposite direction as the loops they overlap (Figure 5D, E, Figure 4—figure supplement 1D, E). These two histone H3 modifications are mutually exclusive and have opposite effects on gene expression, marking enhancers and silencers, respectively (Pasini et al., 2010). Both modifications are able to impact distal gene expression via chromatin interactions (Cai et al., 2021).

Upregulated genes between MCF10A and MCF10CA1a showed similar epigenetic signatures at both proximal and distal regions, regardless of whether the loop they overlap gets stronger or weaker (Figure 5D). Promoter regions gained H3K27ac, while distal regions gained H3K27ac and lost H3K27me3. There is, however, a significant difference in the extent of distal changes depending on the loop direction, with strengthened loops exhibiting a significantly higher increase in distal H3K27ac and a decrease in H3K37me3 marks. This behavior further supports the notion that these distally looped regulatory regions are important functional elements. Downregulated genes showed distinctly different signatures (Figure 5E). Genes that overlap with weakened loop anchors showed decreased promoter H3K27ac and distal H3K27ac and increased distal H3K27me3, consistent with signatures typically associated with reduced gene expression (Pasini et al., 2010). Interestingly, genes that overlap strengthened loop anchors showed different patterns, with a gain in promoter H3K27ac and loss of distal H3K27me3 repressive marks. We conclude that expression of a subset of progression-associated genes is strongly correlated with loop formation. These trends are similar but weaker for genes that change between different cell types (Figure 4—figure supplement 1D, E).

Taken together, our genome-wide analysis of structural and regulatory changes during MCF10A cancer progression has highlighted hundreds of restructured regions where cancer-relevant genes are differentially regulated. These findings suggest that, while relatively rare, changes in chromatin looping may be associated with regulatory changes that drive expression of hundreds of oncogenes during cancer progression.

## Discussion

Multiple levels of chromatin organization and integration with epigenetic parameters contribute to regulation of gene expression. We have identified dynamic chromatin organizational changes on multiple scales during breast cancer progression using the well-characterized MCF10A model system. By comparing both a pre-malignant and metastatic cell line to non-cancerous epithelial cells we were able to detect both early- and late-stage changes as well as similarities in genome structure and relate them to gene expression changes, including many cancer-relevant genes.

We found that compartmental shifts occur more often in the early stages of cancer development. This behavior is consistent with previous studies that have shown intermixing of chromatin A and B compartments in cancer cells (Johnstone et al., 2020; Rossini et al., 2024). The general shift towards more active compartments during cancer progression may reflect a broader epigenetic landscape and greater heterogeneity in gene expression within cancer cell populations (Corces et al., 2016; Liu et al., 2025; Guo et al., 2019; Carter and Zhao, 2021; Flavahan et al., 2017).

On a finer scale, structural changes in TADs occurred more often during later stages of metastasis. We also found an abundance of weakened TAD boundaries in MCF10 breast cancer progression compared with boundaries that gain insulation. This finding is in line with previous studies, which have shown TAD weakening in triple-negative breast cancer (Kim et al., 2022; van den Brand et al., 2025), but is contrary to observations in prostate cancer, which have suggested the formation of many additional TADs (Taberlay et al., 2016), or findings of minimal changes in TAD structure in colorectal or breast cancer (Johnstone et al., 2020; Rossini et al., 2024). The differences in TAD changes across cancer studies may reflect differences in the types of cancer samples analyzed, or the analysis methods used. For example, we detected subtle changes in IS at TAD boundaries in the MCF10 model, while other methods tailored toward detecting more drastic restructuring events, such as the formation of TAD cliques or TAD fusions, found different behaviors (Rossini et al., 2024; Choppavarapu et al., 2025). Recent studies comparing chromatin structures across patient samples have also revealed a high degree of TAD and loop heterogeneity among tumors, even of the same cancer type, and it is thus not surprising that patterns observed between different cancer types and in a cell culture model would vary even further (Choppavarapu et al., 2025). The weakened TAD boundaries in the MCF10 progression system are typically strong boundaries in cancer types, suggesting that the same boundaries are not weakened universally in cancer. The weakening of TAD boundaries is intriguing in that it may point to higher heterogeneity in chromatin structure in more aggressive cancer stages, possibly contributing to more extensive gene misregulation (Liu et al., 2025; van den Brand et al., 2025; Choppavarapu et al., 2025). Further studies using single-cell analysis in the MCF10CA1a population will be necessary to confirm if this is the case.

Comparing the structural and functional features of the MCF10 progression system to other breast cancer datasets revealed many common chromatin loops, TADs, and differentially expressed genes, as well as differences between various cancer progression models and patient data. There are hundreds of genes that change over the course of MCF10 progression that are also significantly different in breast cancer tumors from patients compared to healthy controls, including many that overlap with chromatin loops; however, this represents only roughly a quarter of all differential genes in the MCF10 progression model. Despite this transcriptional diversity, chromatin loops and TAD boundaries detected in MCF10 cell lines showed similar average signatures in mammary epithelial cell lines and triple-negative breast cancer patients. However, cell type-specific differences do appear among differential features based on their timing and direction of change in the MCF10 progression model. For example, loops that are stronger in metastatic MCF10CA1a cells were also significantly stronger than static MCF10 loops among TNBC patients, making them a particularly interesting subset of regions to explore further.

Chromatin loops functionally connect gene promoters to distal regulatory elements. In the MCF10 model, many genes differentially regulated during cancer progression are associated with chromatin loops shared between all three cell lines, but which show changes in distal enhancer H3K27ac and H3K27me3. These trends were stronger with upregulated genes, suggesting that we may need to explore different epigenetic signatures to better understand how chromatin structure may influence repression. We also observed that more highly expressed genes are more likely to overlap with chromatin loops. ABC analysis showed that looped enhancer–promoter pairs also exhibit greater correlation between distal enhancer H3K27ac and gene expression than non-looped enhancers due to their increased contact frequency. These findings suggest that persistent chromatin loops that do not change during cancer progression nevertheless have functional relevance and that they do so by bridging enhancers to target gene promoters.

ABC analysis also revealed that subtle changes in chromatin contact can contribute to the rewiring of enhancer–promoter regulatory connections. Enhancer–promoter pairs that exhibit changes in contact correlated with stronger changes in target gene expression than those with only changes in activity, in line with the concept that contact with distal regulatory elements is an important component of gene regulation (Yang and Hansen, 2024). We also found that changes in chromatin contact are associated with more modest changes in activity, and vice versa. This correlation between enhancer activity and enhancer–promoter contact further points to a functional link between the two. Together, these results suggest that both contact frequency and activity contribute to enhancer–promoter connectivity.

Strong changes in contacts at chromatin loops are relatively rare across cancer progression, but many clear examples do exist, and they are notably enriched for differentially expressed genes. Interestingly, only a small portion of differential loops can be explained by changes in CTCF binding at anchors, suggesting other forces may be influencing their contact frequency. Loss of H3K27ac can be seen at the anchors of lost loops, consistent with the idea that active enhancers can help recruit cohesin to chromatin (Georgiades et al., 2023; Rinzema et al., 2022). Therefore, some weakened loops might be explained by a loss of H3K27ac, which leads to a loss of cohesin at that region.

We also found that genes at differential loops are more likely to be differentially regulated in the same direction as the change in loop strength. This supports the notion that a subset of genes may be regulated in a structure-dependent manner (Choppavarapu et al., 2025; Reyes-Gopar et al., 2025; Yost et al., 2025). This interpretation is in line with previous observations, which have shown a subset of genes to be sensitive to cohesin or NIPBL depletion, which disrupts chromatin loops (de Wit and Nora, 2023; Chakraborty et al., 2024; Xiao et al., 2021; Stolper et al., 2023; Cuartero et al., 2018; Guckelberger et al., 2024; Kubo et al., 2021; Thiecke et al., 2020). Importantly, these findings suggest this subset of structure-sensitive genes may include many with relevance to breast cancer progression.

Epigenetic signatures at gene promoters and distal regions differed based on the direction of gene change. Upregulated genes consistently showed a gain of active-associated H3K27ac marks at both promoters and distal regions, and a loss of distal repressive H3K27me3 marks. These changes were shared between static, strengthened, and weakened loops, although upregulated genes at strengthened loops had stronger distal changes. These findings are consistent with loops functionally supporting interactions with distal enhancers via increased contact. However, downregulated genes showed more complex patterns. Fewer downregulated genes could be explained by changes in H3K27ac or H3K27me3 at the promoter or distal regions, with over half of downregulated genes having no clear epigenetic driver, compared to only 15% of upregulated genes. This suggests that these histone marks are not sufficient to explain downregulated genes as well as they can explain upregulated genes. Downregulated genes at differential loops also showed opposite patterns based on the direction of loop change; weakened loops showed loss of distal H3K27ac and gain of H3K27me3 consistent with an inactivated enhancer, while strengthened loops showed the opposite. Additional studies will be required to fully understand the repressive mechanisms in this system and how they relate to chromatin structure.

Our study has several limitations. While the MCF10 progression system is a well-established and -characterized model, it does not reflect the complexities of in vivo tumor formation. In addition, the MCF10 cell lines used here do not fully represent all stages of cancer progression as they encompasses normal, pre-malignant, and metastatic states, but lack a separate malignant but non-metastatic state such as MCF10DCIS. Furthermore, our analysis is correlative in nature and does not demonstrate causality of chromatin structure changes at the loci of differentially expressed genes. Follow-up studies exploring any of the gene loci identified here as possibly being contact-dependent could help elucidate these relationships. Additionally, as a population-level assay, Micro-C is only able to provide aggregate data across an entire population of cells. To address how cell-to-cell heterogeneity contributes to some of the functional relationships we observe, and whether that heterogeneity is occurring at a cellular or population level, we would need to apply single-cell sequencing or imaging-based approaches. These questions will be the subjects of future studies.

In summary, using the MCF10 breast cancer model, we have generated a rich genomic dataset of structural and functional changes in the genome during breast cancer progression. Our data uncovers new insights into the structure-function relationship in gene regulation and into the role of genome organization during malignant breast cancer progression.

## Materials and methods

**Key resources table:** 

Reagent type (species) or resource	Designation	Source or reference	Identifiers	Additional information
Cell line (*Homo sapiens*)	Cell line MCF10A	ATCC	Cat#:CRL-10317; RRID:CVCL_0598	Non-cancerous mammary epithelial cells
Cell line (*Homo sapiens*)	Cell line MCF10AT1	Karmanos Cancer Institute	ID:MCF10AneoT; RRID:CVCL_5554	Pre-malignant mammary epithelial cells
Cell line (*Homo sapiens*)	Cell line MCF10CA1a	Karmanos Cancer Institute	ID:MCF10CA1a.cl1; RRID:CVCL_6676	Metastatic mammary epithelial cells
Antibody	anti-human CTCF (rabbit monoclonal)	Cell Signaling Technology	Cat#:3418	ChIP-Seq (20 µl per 150 µg chromatin)
Antibody	anti-human H3K27ac (rabbit polyclonal)	AbCam	Cat#:ab4729; RRID:AB_2118291	ChIP-Seq (2 µg per 10 µg chromatin)
Antibody	anti-human H3K27me3 (mouse monoclonal)	AbCam	Cat#:ab6002; RRID:AB_305237	ChIP-Seq (4 µg per 15 µg chromatin)
Commercial assay or kit	Dovetail Micro-C Kit & Assay Service	Dovetail Genomics	Cat#:20101E	Loop level project
Commercial assay or kit	Direct-zol RNA Miniprep	Zymo Research	Cat#:R2050	
Commercial assay or kit	NEBNext rRNA Depletion Kit v2	New England Biolabs	Cat#:7400	
Commercial assay or kit	NEBNext Ultra II RNA-Seq Library Prep Kit	New England Biolabs	Cat#:E7770	
Software, algorithm	BWA mem	PMID:19451168	RRID:SCR_010910	Version 0.7.17
Software, algorithm	Pairtools	https://doi.org/10.1101/2023.02.13.528389	RRID:SCR_023038	Version 0.3.0
Software, algorithm	samtools	PMID:33590861	RRID:SCR_002105	Version 1.17
Software, algorithm	Juicer tools	PMID:27467249	RRID:SCR_017226	Version 1.22.01
Software, algorithm	NeoLoopFinder	PMID:34092790		
Software, algorithm	CALDER	PMID:33972523		Version 2.0
Software, algorithm	FAN-C	PMID:33334380		Version 0.9.21
Software, algorithm	SpectralTAD	PMID:32689928		Version 1.18.0
Software, algorithm	SIP	PMID:32127418		Version 1.6.2
Software, algorithm	STAR	PMID:23104886	RRID:SCR_004463	Version 2.4
Software, algorithm	Trim Galore	https://doi.org/10.5281/zenodo.5127898	RRID:SCR_011847	
Software, algorithm	Bowtie2	PMID:22388286	RRID:SCR_016368	
Software, algorithm	MACS2	PMID:18798982	RRID:SCR_013291	
Software, algorithm	DESeq2	PMID:25516281	RRID:SCR_015687	Version 1.42.0
Software, algorithm	CSAW	PMID:26578583	RRID:SCR_026738	
Software, algorithm	GEPIA	PMID:40396370	RRID:SCR_028242	
Software, algorithm	GenomicRanges	PMID:23950696	RRID:SCR_000025	
Software, algorithm	mariner	PMID:38814811		
Software, algorithm	plotgardener	PMID:35134826		
Software, algorithm	Activity-by-contact	PMID:31784727		
Software, algorithm	nullranges	PMID:37084270		
Software, algorithm	GSEA	PMID:16199517	RRID:SCR_003199	
Software, algorithm	HOMER	PMID:20513432	RRID:SCR_010881	

### Cell culture

MCF10A cells were obtained from AATC (CRL-10317). MCF10AT1 (MCF-10AneoT) and MCF10CA1a (MCF10CA1a.cl1) cells were obtained from the Karmanos Cancer Institute. All cells were received frozen, thawed in media, and grown to a confluence of 80% after two to five passages. Stock solutions were frozen down to be used for all experiments. The identity of each cell line has been authenticated using short tandem repeat profiling and cell lines were tested periodically for the absence of mycoplasma.

MCF10A and MCF10AT1 cells were cultured in standard high calcium medium with growth factors, consisting of DMEM/F12 (Invitrogen, 21041025) supplemented with 1.05 mM CaCl_2_, 10 mM HEPES, 10 µg/ml insulin (Sigma, I1882), 20 ng/ml EGF (Peprotech, AF-100-15), 0.5 µg/ml Hydrocortisone (Sigma, H0135), 100 ng/ml cholera toxin (Sigma, C8052), and 5% horse serum (Invitrogen, 16050122). MCF10CA1a cells were cultured in standard high calcium medium without growth factors, consisting of DMEM/F12, 1.05 mM CaCl_2_, 10 mM HEPES, and 5% horse serum.

### Karyotypic analysis

Metaphase chromosomes were prepared by incubating cells with 100 ng/ml Colcemid (Roche, Brighton, MA) for 2 hr, followed by mitotic shake-off. The mitotic cells were then treated with a hypotonic solution (0.075 M KCl) for 20 min at 37°C. After this treatment, the cells were centrifuged, the supernatant was extracted, and the cells were initially fixed with a methanol/acetic acid solution (3:1). This step was repeated three times. Finally, the cells were fixed onto a slide using a humidity-controlled Thermotron (Thermotron, Holland MI).

SKY probes were purchased from Applied Spectral Imaging (Carlsbad, CA) and hybridized onto slides that were aged at 37°C for 3 days. Detection was then carried out according to the protocol provided by Applied Spectral Imaging, using the following purchased antibodies: Avidin Cy5 (Rockland Immunochemicals, Limerick, PA), Mouse Anti-Digoxin antibody (Sigma-Aldrich), and a goat anti-mouse antibody conjugated to Cy5.5 (Rockland Immunochemicals). Slides were then mounted and counterstained with DAPI (Vector Laboratories, Newark, CA). Hybridization occurred over a period of two days at 37°C. Approximately 20–25 metaphases were imaged and karyotyped using the ASI GenASIS 8.2.2 software on an Olympus BX63 microscope (Evident, Tokyo, Japan) equipped with a Spectral Cube (Applied Spectral Imaging, Carlsbad, CA).

Composite karyotypes for the three cell lines, listing abnormalities found in three or more metaphases, are as follows:

**MCF10A**: 41–53, XX, idic(1)(q10), +1, der(3)t(3;9)(p21;p22), i(8)(q10), der(9)t(3;5;9)(p14;p23;q34;p21)
**MCF10AT1**: 42–49, XX, der(3)t(3;9)(p21;p22), t(3;17)(p14;p11.2), t(6;19)(p25;q12q13.3), der(9)t(3;5;9)(p14;p23;q34;p21), t(19;9)(q34;p11), +20
**MCF10CA1a**: 43–63, XX, der(3)t(3;9)(p21;p22), +der(3)t(3;9)(p21;p22), t(3;17)(p14;p11.2), der(9)t(3;5;9)(p14;p23;q34;p21), +10

### Micro-C library preparation

Cells were frozen and pellets of 1 M cells were used for Micro-C library preparation. The Micro-C library was prepared using the Dovetail Micro-C Kit according to the manufacturer’s protocol. Briefly, the chromatin was fixed with 0.3 M disuccinimidyl glutarate and 37% formaldehyde in the nucleus. The cross-linked chromatin was then digested in situ with micrococcal nuclease (MNase). Following digestion, cells were lysed with 20% SDS to extract the chromatin fragments, and the chromatin fragments were bound to Chromatin Capture Beads. Next, the chromatin ends were repaired and ligated to a biotinylated bridge adapter followed by proximity ligation of adapter-containing ends. After proximity ligation, the crosslinks were reversed, the associated proteins were degraded, and the DNA was purified, then converted into a sequencing library using Illumina-compatible adaptors. Biotin-containing fragments were isolated using streptavidin beads prior to PCR amplification. Each library was sequenced on an Illumina Novaseq platform to generate 240–340 million 2 × 150 bp read pairs (average depth of 280 M reads). Micro-C libraries were prepared by Dovetail Genomics (Scotts Valley, CA). Four pellets and subsequent Micro-C libraries (technical replicates) were prepared per culture of cells (biological replicates), with two biological replicates grown per cell line on separate weeks.

### RNA-Seq library preparation

Total RNA was isolated from cells using Trizol (Life Technologies) and purified using the Direct-zol RNA kit (Zymo Research, Irvine, CA, USA: R2050). RNA quality and quantity were assessed using the RNA 6000 Nano Kit with the Agilent 2100 Bioanalyzer (Agilent Technologies, Santa Clara, CA). RNA quantity was further assessed using a Nanodrop2000 (Thermo Scientific, Lafayette, CO) and Qubit HS RNA assay (Thermo Fisher Scientific). Total RNA was depleted of ribosomal RNA (New England Biolabs, NEB #7400), reverse transcribed using a random hexamer strategy, and strand-specific adapters were added following the NEBNext Ultra II RNA-seq library prep kit (New England Biolabs, E7770). Paired-end sequencing was used to generate high depth coverage ranging from 80 to 120 million paired-end reads. Three biological replicates were generated per cell line from independent cultures and library preparations.

### ChIP-Seq library preparation

ChIPseq for CTCF (Cell Signaling Technology, catalog number 3418) and histone marks H3K27ac (Abcam, ab4729) and H3K27Me3 (Abcam ab6002). Independent biological replicates of each cell line (MCF10A, MCF10AT1, and MCF10CA1a) were performed as described, including the optional step of snap freezing of chromatin and excluding the optional third washing step (O’Geen et al., 2010). Additionally, the chromatin was precleared with protein A/G beads for 2 hr at 4°C prior to incubation with antibodies. For CTCF, we used 20 µl of antibody (Cell Signaling Technologies, 3418S) and 150 µg of chromatin for each sample. For H3K27ac, 10 µg of chromatin was used and 2 µg of antibody, while for H3K27me3, 15 µg of chromatin was used with 4 µg of antibody. Two biological replicates were generated per cell line from independent cultures and library preparations.

### ATAC-Seq library preparation

The OMNI-ATACseq protocol was followed as previously described, with an optimized 5 min of nuclear extraction rather than 3 min (Maor-Nof et al., 2021; Corces et al., 2017). Two biological replicates were generated per cell line from independent cultures and library preparations.

### Micro-C processing

Micro-C data from each technical replicate (library) was processed from raw reads into contact maps using guidelines outlined by Dovetail Genomics (Dovetail, 2025). Paired reads were aligned to the hg38 human genome assembly (NCBI GRCh38) using BWA mem (version 0.7.17; settings: -5SP -T0) (Li and Durbin, 2009). Pairtools (version 0.3.0) was then used to parse ligations, sort reads, and remove PCR duplicates using the parse (settings: --min-mapq 40 --walks-policy 5unique --max-inter-align-gap 30), sort, and dedup (settings: --mark-dups) commands (Abdennur et al., 2023). Alignment files were generated using pairtools split to create .pairs and .sam files, and samtools view (version 1.17; settings: -bS) to create .bam files (Danecek et al., 2021). Final .bam alignment files were sorted and indexed using samtools sort and index commands. The .bam files were used to extract QC metrics using a script from Dovetail Genomics, and calculate complexity using preseq lc_extrap (settings: -bam -pe -extrap 2.1e9 -step 1e7 -seg_len 1000000000). Pairs files were used to generate contact maps using juicer_tools pre (version 1.22.01), and normalized using SCALE (Durand et al., 2016).

For merged contact maps, we first merged pairs files using pairtools merge, and then ran juicer_tools pre command on the resulting .pairs files. In total, we generated contact maps for each library (technical replicates), each sample (biological replicates), each cell type, and we created one fully merged map including all reads for a precise map of shared features. Micro-C maps for individual technical replicates and cell types are available on GEO (accession GSE254045).

### Micro-C copy number variation analysis

SV and copy number variations were detected from contact maps using NeoLoopFinder calculate-cnv at 1-Mb resolution (-e uniform; Supplementary file 2; Wang et al., 2021). These values were averaged over the course of large-scale variations and used as correction factors for differential loop analysis (see Differential loop, TAD, gene, and peak analysis).

### Regions excluded in analysis

Denylists of regions to ignore for feature calling were generated based on regions where SCALE normalization factors were unable to be calculated at 5 or 10 kb, ignoring single bins and merging continuous areas within 100 kb. ABC analysis also combined this denylist with the ENCODE denylist for hg38 (Amemiya et al., 2019).

### Micro-C compartment calling

Compartments were called using CALDER (version 2.0; R version 4.2.1) at 10-kb resolution and SCALE normalization (Liu et al., 2021). We also used FAN-C (version 0.9.21) to calculate eigenvectors at 100 kb using SCALE normalization to build saddle plots, and oriented eigenvectors manually based on overlap with active genes (Kruse et al., 2020). A table of subcompartments for each cell type is available on GEO (accession GSE254045).

### Micro-C topologically associated domain calling

Topologically associated domains (TADs) were called using SpectralTAD (version 1.18.0) at 10-kb resolution with SCALE normalization, a window size of 300, and a level of 3, with a quality filter applied (Cresswell et al., 2020). We then created a unified TAD list by merging TADs that overlap at both ends between cell types, with a maximum gap of 10 kb (1 pixel). We then combined this analysis with continuous IS calculations from FAN-C insulation command at 10-kb resolution with SCALE normalization. We then used the FAN-C boundaries command to detect IS boundaries and only kept TADs that also overlapped with an IS boundary. Tables of TADs and boundaries are included in Supplementary files 4 and 5. IS tracks for each cell type are available on GEO (accession GSE254045).

### Micro-C chromatin loop calling

Chromatin loops were called using SIP (version 1.6.2), run at 5 and 10 kb and then merged to 10 kb (-g 2 -fdr 0.05). Loops were called in each cell type map using SCALE normalization, in addition to the combined map, and then merged similarly to TADs to create a unified loop list (Supplementary file 3; Rowley et al., 2020). Cell type loops are available on GEO (accession GSE254045).

### RNA-Seq processing

All RNA-Seq data was analyzed using the nf-core/rnaseq pipeline (Patel et al., 2020). Adapter and quality trimming was performed with Trim Galore (Babraham Bioinformatics, 2017). Reads were aligned to the hg38 reference genome using STAR (Dobin et al., 2013), and gene expression was quantified using Salmon (Patro et al., 2017). Differentially expressed genes were called using DESeq2 (Love et al., 2014). An adjusted p-value of 0.05 and a log_2_ fold change of 1 were used as thresholds to select significant differential expression. A table of all differentially expressed genes and their DESeq2 metrics are listed in Supplementary file 6. A full table of expression data for all genes can be found on GEO (accession GSE320216).

### ChIP-Seq processing and peak calling

ChIP-Seq data for CTCF, H3K27ac, and H3K27me3 was processed as detailed previously (Fritz et al., 2018; Ghule et al., 2023). In summary, adapters were cut (cutadapt v1.11), and low-quality reads trimmed (Galaxy FASTQ Quality Trimmer 1.0.0; window 10, step 1, minimum quality 20). Reads were mapped to the human genome (hg38 canonical) using STAR version 2.4 (Dobin et al., 2013) with splicing disabled (–alignIntronMax 1). Enriched regions (narrowPeak calls for CTCF and H3K27me3, broadPeak calls for H3K27ac) for each replicate were generated using MACS2 (Feng et al., 2012), and replicates were then evaluated using deepTools (Ramírez et al., 2016) to correlate alignments and IDR (Li et al., 2011) to evaluate peak call reproducibility. After pooling replicates, MACS2 (Zhang et al., 2008) was used to call peaks at high stringency (p-value <10e−5); these peaks were further filtered according to IDR cutoffs. ChIP-Seq data is available on GEO (accession GSE98551 for CTCF, and GSE229295 for H3K27ac and H3K27me3).

### ATAC-Seq processing and peak calling

Read trimming and quality filtering was performed using FastQC (Bioinformatics, 2015) and TrimGalore (Babraham Bioinformatics, 2017). The filtered fastq were then downsampled to approximately 50 million reads per replicate. Reads were aligned to the hg38 reference genome using Bowtie2 (Langmead and Salzberg, 2012). Mitochondrial, multi-mapped, and low-quality reads, and duplicates were removed using samtools (Danecek et al., 2021). MACS2 (Zhang et al., 2008) was used to call narrowPeaks, followed by IDR (Li et al., 2011) to generate sample level peak sets. ATAC-Seq data is available on GEO (accession GSE320215).

### Enhancer and promoter definitions

Gene promoters were defined as regions between 2000 bp upstream and 500 bp downstream of gene transcription start sites.

Potential enhancer regions were identified based on regions that contained both an ATAC-Seq and H3K27ac ChIP-Seq peak. For ABC analysis, potential enhancers were defined as 150,000 ATAC-Seq peaks with the highest levels of H3K27ac signal, but were subset for regions with H3K27ac peaks after running (see below).

### Compartmental saddle plots

Saddle plots were made manually in R. We selected three chromosomes that had no major karyotypic differences between cell lines and had high correlation of eigenvectors between replicates with the same signage (chr2, chr12, and chr17). For each chromosome and cell type, we sorted eigenvectors into 20 bins. We then calculated the mean observed/expected values (using SCALE normalization) between regions belonging to different bins and plotted it as a heatmap.

### Differential loop, TAD, gene, and peak analysis

Differential genes were calculated using DESeq2 (version 1.42.0) (Love et al., 2014). Each cell type had three replicates, and a design of ~cellType was used. No fold-change cutoff was applied. Genes with an adjusted p-value below 0.01 were considered significant.

Differential H3K27ac within ATAC-Seq peaks were calculated using a similar design, but with a p-value cutoff of 0.05.

Differential CTCF peaks were called using the CSAW package following the workflow described in the original publication (Fritz et al., 2018), and an adjusted p-value cutoff of 0.1.

Differential loops were also identified using DESeq2, but with additional adjustments. Raw, un-normalized loop counts were pulled from each technical replicate map, resulting in 8 values per cell type (4 technical replicates for each of 2 biological replicates per cell type). An LRT design was used of ~technicalRep + biologicalRep + cellType, with ~technicalRep + biologicalRep as the comparison. Size factors were provided manually based on NeoLoopFinder output (Supplementary file 2). Regions of duplication were identified based on NeoLoopFinder CNV output and spectral karyotypes. For each duplicated region, the CNVs across the region were averaged and used as custom size factors to correct for differences in chromosomal copy number between cell lines. Differential loops were identified based on a fold-change cutoff of 1.5 and an adjusted p-value cutoff of 0.025.

Differential TAD boundaries were detected using an alternative method. ISs were pulled from all TAD boundaries at the technical replicate level (8 values per cell type). A *T*-test was then applied for each pairwise comparison of cell types, and p-values were adjusted using FDR. Boundaries with an adjusted p-value below 0.01 were considered significant.

Differential loops, TAD boundaries, and genes were clustered based on their patterns of change across the three cell types using *k*-means clustering of centered and scaled normalized counts.

### Integration of Cancer Genome Atlas data

RNA-Seq data from the breast cancer (BRCA) cohort of the Cancer Genome Atlas was used to analyze whether genes differentially expressed in the MCF10 progression model are also differentially expressed in breast cancer tumor tissues (*n* = 1102) from patients compared to normal control tissues (*n* = 113). Normalized gene transcript counts from RSEM were downloaded from Xena (Goldman et al., 2020). Protein coding genes that are differentially expressed between tumors and paired peritumors in TCGA-BRCA data were identified using GEPIA3 using DESeq2 with a log_2_ fold-change cutoff of 1 and an adjusted p-value cutoff of 0.05. A total of 5289 differential genes were identified from the TCGA-BRCA data: 3168 with significantly higher expression in tumor samples, and 2121 with significantly higher expression in normal samples (Tang et al., 2017). Overall survival curves for select genes were generated using GEPIA (Tang et al., 2017).

### Integration of additional breast cancer structural data

Chromatin loops and TAD boundaries identified in the MCF10 progression model were also examined in other triple-negative breast cancer cell lines and patient samples using data from GEO (accession GSE167150) (Kim et al., 2022). IS tracks were calculated as for MCF10 cell lines, using FAN-C at 10-kb resolution and KR normalization. Chromatin loop counts were extracted using KR normalization at 10-kb resolution.

### Micro-C feature overlap and aggregate analysis

Micro-C feature overlap and analysis was conducted in R primarily using the *GenomicRanges*, *InteractionSet*, and *mariner* packages (Lawrence et al., 2013; Lun et al., 2016; Davis et al., 2024). To overlap chromatin loops with other features such as promoters and enhancers, we used loop anchors at 10-kb resolution and allowed features within 10 kb (±1 Hi-C pixel) of the loop anchors to be considered overlapping. We used this broad definition for loop overlap to account for the fact that loops are non-punctate and show increased contact frequency within several bins on either side of the called loop pixel, as evidenced by both individual loci and aggregate loop analysis. Aggregate matrices of loop pixels and TAD boundaries were generated using *mariner* and visualized using *plotgardener* (Davis et al., 2024; Kramer et al., 2022).

### Activity-by-contact

The ABC was used based on Fulco et al., 2019 with slight adjustments (Fulco et al., 2019). To allow for direct comparison of all enhancer–promoter pairs across cell lines, we manually defined potential enhancer regions and used the same input for each cell type. These potential enhancer regions were defined as they typically are in the pipeline, by finding 150,000 ATAC-Seq peaks with the highest H3K27ac levels. The output was filtered using a suggested ABC score cutoff of 0.025 to identify likely enhancer–promoter pairs. To allow for direct comparison with our other enhancer analysis, we filtered the output based on the enhancer regions also overlapping H3K27ac, which still represented a majority of the valid pairs identified.

### Matched enhancer–promoter sets

Covariate-matched subset selection among non-looped enhancer–promoter pairs was performed using the *matchRanges* function from the *nullranges* package (Love M Davis et al., 2022; Kolberg et al., 2020). Enhancer–promoter pair distance or mean Micro-C contact frequency were used as covariates. Matching was done with the stratified matching method without replacement.

### Gene ontology and gene set enrichment analysis

Gene ontology term enrichment was performed in R using the *gprofiler2* package (Kolberg et al., 2020). Gene set enrichment analysis (GSEA) was performed with the *GSEA* software, using size factor normalized RNA-Seq counts as input (Subramanian et al., 2005) and the Hallmark H1 gene set.

### ATAC-Seq motif analysis

Motif analysis of ATAC-Seq peaks within strengthened and weakened loop anchors was conducted using the HOMER suite findMotifsGenome.pl script with size given (Heinz et al., 2010). ATAC-Seq peaks within the anchors of static loop anchors were used as background.

### H3K27ac peak pileup analysis

H3K27ac ChIP-Seq analysis in the anchors of gained, lost, and static loops was conducted using deeptools (Ramírez et al., 2016). Alignment files were normalized using RPGC with the bamCoverage function, and adjusted using scale factors generated from edgeR TMM normalization factors of counts from overlapping H3K27ac and ATAC-Seq peaks (Robinson et al., 2010). Aggregate profile plots were then created using the plotProfile command.

### Genomic data visualization

Micro-C contact frequency maps, aggregate analysis plots, gene annotations, and genomic signal tracks (RNA-Seq, ChIP-Seq, and ATAC-Seq) were visualized and plotted in R using the *plotgardener* package (Kramer et al., 2022).

## Data Availability

The Micro-C, ATAC-Seq, and RNA-Seq datasets generated and analyzed during the current study are available on GEO (SuperSeries GSE320319; SubSeries GSE254045, GSE320215, GSE320216). CTCF ChIP-Seq data was previously published under GEO accession GSE98551. H3K27ac and H3K27me3 ChIP-Seq data were previously published under GEO accession GSE229295. The code used to generate figures from the current study is available on GitHub at the following repository: https://github.com/ksmetz/MCF10-MicroC copy archived at Reed, 2025. The following datasets were generated: Metz ReedKS MisteliT FritzA GreenyerH FrietzeS SteinJ SteinG 2026Genome reorganization and its functional impact during breast cancer progressionNCBI Gene Expression OmnibusGSE32031910.7554/eLife.108135PMC1327174342300122 MetzRKS MisteliT FritzA GreenyerH FrietzeS SteinJ SteinG 2025Genome reorganization and its functional impact during breast cancer progression [Micro-C]NCBI Gene Expression OmnibusGSE25404510.7554/eLife.108135PMC1327174342300122 ReedKS MisteliT FritzA GreenyerH FrietzeS SteinJ SteinG 2026Genome reorganization and its functional impact during breast cancer progression [ATAC-Seq]NCBI Gene Expression OmnibusGSE32021510.7554/eLife.108135PMC1327174342300122 ReedKS MisteliT FritzA GreenyerH FrietzeS SteinJ SteinG 2026Genome reorganization and its functional impact during breast cancer progression [RNA-Seq]NCBI Gene Expression OmnibusGSE32021610.7554/eLife.108135PMC1327174342300122 The following previously published datasets were used: FritzA BoydJ TyeC LianJ SteinG 2017Chromatin structure and CTCF across the MCF10 breast cancer progression series (ChIP-Seq)NCBI Gene Expression OmnibusGSE98551 SteinG BoydJR GhuleP FrietzeS 2023Long-range genomic contacts and spatiotemporal chromatin landscape of human histone gene clusters at Histone Locus Bodies during the cell cycle in breast cancer [ChIP-seq]NCBI Gene Expression OmnibusGSE229295

## Additional files

Supplementary file 1. Micro-C library quality metrics for individual technical replicates, biological replicates, and cell types.

Supplementary file 2. CNVs from NeoLoopFinder (100-kb resolution). Columns indicate bin coordinates (A–C), and CNV values for MCF10A (D), MCF10AT1 (E), and MCF10CA1a (F).

Supplementary file 3. Chromatin loop summary table for total loop set (*n* = 29,205). Columns include loop anchor coordinates (A–J), loop call status by cell type (K–N), SIP AP score (O), loop name (P), loop span length (Q), average and maximum unnormalized counts (R–S), log_2_ fold-change (T–V), adjusted p-value (W), differential status by pairwise comparison (X–Z), unnormalized counts by technical replicate (AA–AX), maximum log_2_ fold-change (AY), pairwise comparison with greatest fold-change (AZ), differential cluster (BA), *Z*-score normalized counts (BB–BE), and variance stabilized counts (BF–BH).

Supplementary file 4. TAD boundary summary table for total boundary set (*n* = 17,097). Columns include boundary coordinates (A–C), insulation scores by technical replicate (D–AA), adjusted p-values, difference in insulation score, and differential status for each pairwise comparison (AB–AJ), differential status across all comparisons (AK), differential cluster (AL), average insulation score per cell type (AM–AO), and *Z*-score normalized insulation scores (AP–AS).

Supplementary file 5. TAD summary table for total set of TADs (*n* = 13,231). Columns include TAD boundary coordinates (A–J), and whether the TAD was called in each cell type (K–M).

Supplementary file 6. Differentially expressed genes from the MCF10 progression series (*n* = 8840). Columns indicate gene coordinates (A–E), gene identifiers (F–I), base mean expression (J), pairwise log_2_ fold-change (K–M), adjusted p-values (N–P), and differential status (Q–S), differential cluster (T), unnormalized counts by replicate (U–AC), *Z*-score normalized counts (AD–AF), and variance stabilized counts (AG–AI).

MDAR checklist
