# Supplementary material for: Genome reorganization and its functional impact during breast cancer progression
Source: eLife. 2026 Jun 16;14:RP108135. doi: 10.7554/eLife.108135 (PMC13271743; doi:10.7554/eLife.108135)
Supplement: Peer review history

# Editor report: eLife Assessment

**DOI:** 10.7554/eLife.108135.3.sa0

**Authors:** Yu Zhao

**Affiliations:** Institute of Radiation Medicine, Chinese Academy of Medical Sciences and Peking Union Medical College China

**Keywords:** Compelling, Fundamental

The study by Reed et al. provides **fundamental** findings defining the topological changes that occur during tumorigenesis. These **compelling** findings enhance the understanding of stable long-range connections among genes that reprogram cancer-related functions.

# Referee report: Reviewer #1 (Public review)

**DOI:** 10.7554/eLife.108135.3.sa1

Summary:

In their manuscript, Metz Reed and colleagues present an exceptionally thorough analysis of three-dimensional genome reorganization during breast cancer progression using the well-characterized MCF10 model system. The integration of high-resolution Micro-C contact maps with multi-omics profiling provides compelling insights into stage-specific dynamics of chromatin compartments, TAD boundaries, and looping events. The discovery that stable chromatin loops enable epigenetic reprogramming of cancer genes while structural changes selectively drive metastasis-associated pathways represents a significant conceptual advance. This work substantially deepens our understanding of genome topology in malignancy.

Strengths:

This work sets a benchmark for integrative 3D genomics in oncology. Its methodological sophistication and conceptual advances establish a new paradigm for studying nuclear architecture in disease.

Comments on revised version:

The authors made a significant effort to improve the manuscript. My comments were sufficiently addressed.

# Referee report: Reviewer #2 (Public review)

**DOI:** 10.7554/eLife.108135.3.sa2

Using the MCF10 breast cancer progression sequence, the authors combined high-resolution Micro-C chromatin conformation capture with RNA-seq and ChIP-seq to depict the sequential reorganization of compartments, topologically associated domains (TADs), and long-range loops in benign, pre-tumor, and metastatic states, and coupled these three-dimensional changes with gene expression and enhancer activity. Four main findings were: (i) chromatin structure was largely quiescent, still limiting gene output differentiation, with upregulated sites being most significantly affected; (ii) enhancer-promoter contact strength covariated with transcriptional amplitude; (iii) 127 genes gained expression with increasing chromatin contact; and (iv) progression-related genes acquired altered histone markers in distal enhancers, which remained connected by stable loops. These conclusions are widely accepted and provide strong justification for the publication of this paper.

# Referee report: Reviewer #3 (Public review)

**DOI:** 10.7554/eLife.108135.3.sa3

Summary:

The authors tackle an important problem- that is defining the topological changes that occur during tumorigenesis. To study this, they use an established stepwise cell model of breast cancer. A strength of their study is a careful, robust differential analysis of topological features across each cell state that is presented clearly and rigorously. They define changes in compartmentalization, TAD structure and chromatin looping. Intriguingly, when the authors integrate differential gene expression with chromatin looping, they see that most differentially regulated genes are not involved in loop changes, suggesting that changes in promoter or enhancer chromatin marks may play a bigger role in regulating transcription than differential loops. The differential topology analysis and its integration with transcription is very well done- one of the best versions of this I have read in the 3D genome field! However, the paper is framed largely as a cancer biology study and it teaches us much less about this. I am worried that some of the trends for each topologic feature are not going to be consistent across the pre-malignant-malignant-metastatic spectrum and would like the authors to soften some of their claims a bit regarding how this clarifies our understanding of cancer evolution.

Updated comments on revision:

There are still some issues with this paper. First, it reads descriptively. It is a series of comparisons with limited biologic insight as changes are always seen in genomics and in this case, they're often not tied back to transcription or gene regulation in cancer. Cell lines do not represent cancer faithfully and in this case should not be argued to represent malignant transformation broadly. The authors did not really soften their language as much as I think required. I would caution the authors to further qualify their results in the context of a single, clonal cell line that has undergone stepwise transformation. This is not a patient cohort analysis or frank progression. This matters because there is likely to be much more noise, not pertinent to transformation, in a cell line model. It doesn't negate the validity of the study, but this language should be adjusted appropriately. It was nice to see the authors compare gene expression data from their model to the primary tumor data, however the limited overlap is concerning that at the least patterns of transcriptional regulation in their model are not faithful to primary tumors. If this is the case, it raises concern that the topological changes are also not generalizable to cancer.

The authors declined a number of functional assays to validate their observations (which are purely correlative). And while I see that the burden of extra experiments may be beyond the scope of this study, they must soften their language to justify the observed relationships.

# Author comment: Author response

**DOI:** 10.7554/eLife.108135.3.sa4

**Authors:** Kathleen S Metz Reed, Andrew Fritz, Haley Greenyer, Kerstin Heselmeyer-Haddad, Seth Frietze, Janet Stein, Gary Stein, Tom Misteli

**Affiliations:** National Cancer Institute Bethesda, MD United States; Department of Biochemistry and University of Vermont Cancer Center, University of Vermont Larner College of Medicine Burlington, VT United States; Department of Biochemistry and University of Vermont Cancer Center, University of Vermont Larner College of Medicine Burlington, VT United States; OMICS Technology Facility, Genetics Branch, Center for Cancer Research, National Cancer Institute, National Institutes of Health Bethesda United States; University of Vermont Cancer Center Burlington United States; Department of Biochemistry and University of Vermont Cancer Center, University of Vermont Larner College of Medicine Burlington, VT United States; Department of Biochemistry and University of Vermont Cancer Center, University of Vermont Larner College of Medicine Burlington, VT United States; National Cancer Institute Bethesda, MD United States

The following is the authors’ response to the original reviews.

**Public Reviews:**

**Reviewer #1 (Public review):**

Strengths:

This work sets a benchmark for integrative 3D genomics in oncology. Its methodological sophistication and conceptual advances establish a new paradigm for studying nuclear architecture in disease.

We appreciate the very kind words.

Weaknesses:

Major Issues

(1) Functional tests would strengthen the observed links between structure and gene changes. For example, the COL12A1 gene loop formation correlates with its increased expression. Disrupting this loop using CRISPR-dCas9 at chr6 position 75280 kb could prove whether the loop causes COL12A1 activation. Such experiments would turn strong correlations into clear mechanisms.

We agree that targeted disruption of specific loops such as *COL12A1* will be important for functional validation of the causal relationships between enhancer-promoter loop formation/dissipation and changes in gene expression. However, the intent of our current study was to profile changes in genome organization at a global scale to deduce general features of cancer progression-associated changes in genome organization, rather than to explore specific loop interactions. The current findings are a foundation for more targeted functional follow-up studies.

(2) The H3K27ac looping idea needs deeper validation. Data suggests H3K27ac loss weakens loops without affecting CTCF. Testing how cohesin proteins interact with H3K27acmodified sites would clarify this process. Degron systems could rapidly remove H3K27ac to observe real-time effects. Also, the AP-1 motifs found at dynamic loop sites deserve functional tests. Knocking down AP-1 factors might show if they control loop formation.

We agree that modulating histone modifications or transcription factors would provide insights into the underlying mechanisms driving the changes we observed. However, such studies utilizing degrons or small molecule inhibitors that globally knock down either H3K27ac or specific transcription factors are often difficult to interpret. For example, assessing the role of AP-1 factors, as suggested, would be complicated by the variety of AP-1 proteins. In addition, H3K27ac reduction could inhibit loop strength either directly (i.e. by reducing cohesin recruitment) or indirectly (i.e. by reducing gene expression which could in turn affect loop strength). Parsing out the exact relationships between these features will require extensive follow-up work and falls outside of the scope of the current study.

(3) Connecting findings to patient data would boost clinical relevance. The MCF10 model is excellent for controlled studies. Checking if TAD boundary weakening occurs in actual patient metastases would show real-world importance. Comparing primary and metastatic tumor samples from the same patients could reveal new structural biomarkers. If tissue is scarce, testing cancer cells with added stroma cells might mimic tumor environment effects.

We have leveraged publicly available datasets to link the observations from the progression model to clinical samples. Specifically, we have compared our datasets to chromatin organization data in non-cancerous mammary epithelial cells (HMEC), five cell lines representing distinct cancer subtypes ranging from less (luminal) to more aggressive (triple negative, TNBC), as well as tissue samples from TNBC patients with contralateral normal controls. We explored the conservation of both loops and TADs identified in the MCF10 progression system in each of these maps, paying particular attention to how features that are differential between MCF10 cells differ across other cancer cell types. We observe a high degree of conservation of static loops and TAD boundaries among the cancer samples, as well as some degree of cell-specific changes among loops and boundaries that change during MCF10 progression. These findings are included in Supplemental Figures 3 and 4 and are discussed on page 7.

Minor Issues

(1) Adding a clear definition for static loops would help readers. For example, state that static loops show less than 10 percent contact change across replicates.

Static loops are defined as loops with a fold-change of 1.5 or more between any two MCF10 cell lines and an adjusted p-value of less than 0.025 considering change across biological and technical replicates. This definition is stated on page 6.

(2) In the ABC model analysis, removing promoter regions from the enhancer list would focus results on true long-range interactions.

The ABC model already excludes the promoter of each gene. Only self-promoters are excluded, whereas the model allows promoters of other genes to act as potential long-range enhancers of the target gene. We have added text to make this clear (see page 11).

(3) Briefly noting why this study sees TAD weakening while other cancer types show different patterns would provide useful context.

The biological reason for TAD weakening in the MCF10 model is not known, but neither the mechanism for boundary weakening nor the reason for apparently different behavior amongst cancers is known. We expanded the text on this discussion slightly, but we refrain from making any definitive claims. We do note that differences in the types of cancer studied or the methods used for detecting changes in TADs (i.e. different sensitivities and thresholds for detecting change) could be responsible (see page 15). We also mention that the loss of insulation at many TAD boundaries detected in our study are subtle changes in intensity that could be potentially missed if using methods tailored to find more drastic changes in TAD architecture.

**Reviewer #2 (Public review):**

While the conclusions are broadly supported, methodological and analytical refinements are required.

We appreciate these comments.

(1) Model representativeness. The long-term culture-adapted MCF10 genome harbours extensive aneuploidies and translocations. Validation of key COL12A1/WNT5A loop dynamics in an independent breast-cancer line (e.g., MDA-MB-231, T47D) or in patientderived organoids/PDX models would strengthen generalizability.

Although the generation of Micro-C datasets in additional cell lines is outside of the scope of this study, we used publicly available Hi-C data from triple negative breast cancer (TNBC) progression and patient samples (Kim, Han & Chun et al. 2022) to assess generalizability of the MCF10 model findings. While these maps are lower resolution than the Micro-C maps used in our study, they are of sufficient depth to detect loops at a similar resolution (10 kb). We report these findings in Supplemental Figures 3 and 4 and discuss them on page 7.

We find that chromatin loops and TAD boundaries detected across the MCF10 system are highly conserved across all other mammary epithelial lines studied. Chromatin loops that were more prominent in MCF10AT1 and MCF10CA1a lines were also significantly stronger in TNBC cells. Insulation score boundaries that were weakened in MCF10CA1a showed strong insulation across all cell lines in TNBC. These findings highlight that different model systems indeed have distinct profiles of structural change, just as they have distinct gene expression profiles.

It is worth noting that direct comparison at individual loci is complicated by variations in gene expression profiles between the MCF10 model and the TNBC progression model; for example, *COL12A1* is not significantly upregulated between normal and TNBC tissues in this study (unlike in the TCGA-BRCA data) and is downregulated between HMEC and TNBC cell lines. Regardless, our analysis provides some indication of conserved and divergent features in the various model systems.

(2) The study remains purely correlative; no perturbation experiments are conducted to demonstrate causal roles of chromatin loops on gene expression. CRISPR interference (CRISPR-Cas9-KRAB/HDAC) or enhancer deletion/inversion should be applied to 3-5 pivotal loops (e.g., COL12A1, WNT5A) to test their impact on target-gene expression and cellular phenotypes (e.g., proliferation, migration).

We agree that targeted disruption of specific loops such as *COL12A1* will be important for understanding the causal relationships between enhancer-promoter loop formation/dissipation and changes in gene expression. However, the intent of our current study was to profile changes in genome organization at a global scale to deduce general features of cancer progression-associated changes in genome organization, rather than exploring specific loop interactions. The current findings are a foundation for more targeted follow-up functional studies.

(3) The manuscript lacks integration with clinical datasets. Integrate TCGA-BRCA data to assess whether elevated COL12A1/WNT5A expression associates with overall survival (OS) or distant metastasis-free survival (DMFS)

To assess clinical significance of specific loci, we have queried expression of all differentially expressed genes in the MCF10 progression system among TCGA-BRCA expression data. We summarize our findings in Supp. Fig. 5E and discuss them on page 8.

We found that roughly 25% of genes that change in our model also change significantly in breast cancer, but only roughly half of those genes change in the same direction (i.e. up-regulated in MCF10CA1a vs MCF10A, and up-regulated in tumor vs normal samples). Interestingly, there was a higher degree of directional agreement between latechanging genes (i.e. genes that change in MCF10CA1a compared to MCF10A and MCF10AT1) than early-changing genes (i.e. genes that change in MCF10AT1 and MCF10CA1a compared to MCF10A).

We have also explored the impact of select highlighted genes on overall survival (OS). We present these data in Supp. Fig. 6 and discuss it on page 8. While not all genes showcased in this study have a significant impact on overall survival, most trend in the same direction as their differential expression would suggest (i.e. genes more highly expressed in cancer vs tumor also have a hazard ratio above 1).

**Reviewer #3 (Public review):**

The differential topology analysis and its integration with transcription is very well done- one of the best versions of this I have read in the 3D genome field!

We appreciate the reviewers’ endorsement.

However, the paper is framed largely as a cancer biology study, and it teaches us much less about this. I am worried that some of the trends for each topologic feature are not going to be consistent across the pre-malignant-malignant-metastatic spectrum and would like the authors to soften some of their claims a bit regarding how this clarifies our understanding of cancer evolution.

We agree that the strength of the study lies in its deep mapping of chromatin architecture and the landscape of enhancers and differentially expressed genes, which we hope to use to better understand the relationship between chromatin structure and gene expression, regardless of their cancer relevance. To better relate the findings in the progression system to cancer, we have added new data from direct comparisons of the MCF10 progression system with multiple patient-derived cancer cell lines and cancer tissues. These data are shown in Supp. Fig. 3 and 4 and discussed on p. 7. Regardless, we have softened the claims regarding cancer progression throughout the manuscript.

Weaknesses:

Major Concerns:

(1) The integration of gene expression and chromatin loops is intriguing. The authors' differential analysis, however, omits consideration of genes that are on and simply further upregulated versus genes that transition on/off or off/on. It would be nice to see the authors break out looping patterns for these two different patterns of regulation, as it may be instructive regarding the rules for how EP loops govern transcription.

To address different types of gene expression patterns, we analyzed 108 genes that went from an unexpressed or “off” state (2 or fewer read counts) in one cell line to an expressed “on” state (100 or more read counts) in another, and 111 genes that go from “on” to “high” (1000 or more read counts). We present these data in Supp. Fig. 8 and discuss the findings on page 9. While neither of these genes were enriched for differential loops, a large number overlap with loop anchors. We found a relationship between loop strength and gene expression levels; genes that are more strongly expressed are more likely to overlap with the anchor of a chromatin loop. All gene sets show similar strong trends at distal regulatory regions.

(2) Given the paucity of differential loops at the majority of genes whose expression changes, the authors should examine chromatin subcompartments, as these may associate more with differential transcription.

We present subcompartment analysis in Supp. Fig. 9. Our CALDER compartment calls are qualitative rather than quantitative, so to explore this we examined how compartments change genome-wide and at specific promoters. We show these data in Supp. Fig. 9 and discuss the findings on page 10-11. We see that between any two cell types, a majority of changes occur between closely related subcompartments, i.e. from A.2.2 to A.2.1 (1 step more A-like) or B.1.1 (1 step more B-like). The promoters of differentially expressed genes have minimal subcompartment changes, but genes that shift from on to off have larger changes. Differentially expressed genes with promoters that shift by multiple subcompartments have significant impacts on fold-change, but smaller shifts have minimal impacts on gene expression. In summary, small changes in subcompartments are very common and have little impact on gene expression, while larger changes are infrequent and correlate more strongly with changes in gene expression.

(3) The authors could push their TAD analysis further by integrating it with transcription. Can they look at genes and their enhancers that span these altered boundaries to see if these shifts impact transcription?

We provide this analysis in Supp. Fig. 9. We started, as suggested, by looking at genes with distal enhancers (as determined by the ABC model) that span a single TAD boundary. However, the number of genes that fit this definition was relatively small, so we expanded to look at any genes with promoters in the proximity (50kb) of differential insulation score boundaries, for which we saw the same trends with more robust signal. Our findings are shown in Supp. Fig. 9 and discussed on page 10. We found that genes near weakened boundaries are not enriched for differentially expressed genes, while those near strengthened boundaries are. Comparing the fold-change of genes near strengthened, weakened, and static boundaries showed a significant inverse correlation between boundary strength and gene expression, although effect sizes were small. These results show that changes in TAD boundary insulation have small but noticeable impacts on gene expression.

(4) The progression of cancer critically goes from a benign -> pre-malignant -> malignant -> metastatic series of steps. The AT1 line is described as 'premalignant' and thus the authors' series omits a malignant line. While I think adding such a sample is an unreasonable request at this point (as it would have had to have been studied in 'batch' with these other samples), the authors should acknowledge that they omit this step and spend some time discussing the genetic, morphologic, and phenotypic features for their 3 conditions. The images in Figure 1S aren't particularly useful- they don't tell the reader that these cells are malignant/benign. The karyotypic data are intriguing but not fully analyzed, so it is hard to know what true phenotype these cells represent. For example, malignant means DCIS/invasive carcinoma - so then what does this pre-malignant cell model represent? The described alteration in the AT1 line is a Ras oncogene, so in some sense, the transition to this line really is just +/- Ras. The authors could spend some time thinking about the effects of Ras specifically on the 3D genome.

We have expanded our discussion of the relevance of the MCF10 model on page 4, and the limitations of the model on page 17. The MCF10 progression model has been extensively used by many laboratories, and its properties have been discussed in detail (i.e. Polizzotti et al. 2012). Critically, the MCF10AT1 cell line is the product not only of Ras oncogene expression but then derived from a 100-day-old precancerous lesion that formed a squamous carcinoma in a mouse, and over this time it accumulated additional changes. The MCF10AT1 line is considered pre-malignant as it has accrued critical changes that prepare it for the metastatic transition, but it does not immediately form tumors when injected back into mice. Unlike the MCF10DCIS cell line which is malignant but not metastatic, the more aggressive MCF10CA1a is classified as both malignant and highly metastatic, forming tumors that quickly metastasize to the lungs in mouse xenografts. While both MCF10AT1 and MCF10CA1a are tumorigenic, we acknowledge the lack of a nonmetastatic malignant cell line in the discussion on page 17. We have also provided updated karyotype characterization of the cell lines used in this study in Supp. Fig. 1B and now include full composite karyotypes in the Methods section (page 18).

**Recommendations for the authors:**

**Reviewer #1 (Recommendations for the authors):**

The reviewer’s recommendations are the same as their public review comments. See our response to the review comments above.

**Reviewer #2 (Recommendations for the authors):**

(1) If conditions permit, it is recommended that inclusion of primary human mammary epithelial cells (HMECs) to distinguish immortalisation-specific from malignancy-specific 3D changes.

Micro-C data of equal resolution is not available for HMECs. We have, however, incorporated analysis of publicly available deeply sequenced Hi-C data of HMECs into several figures that explore the conservation of loops and TADs in these cells (Supp. Fig. 3 and 4).

We find that chromatin loops and TAD boundaries detected across the MCF10 system are highly conserved across all other mammary epithelial lines studied. Chromatin loops that were more prominent in MCF10AT1 and MCF10CA1a lines were also significantly stronger in TNBC cells. Insulation score boundaries that were weakened in MCF10CA1a showed strong insulation across all cell lines in the TNBC system. These findings highlight that different model systems indeed have distinct profiles of structural change, just as they have distinct gene expression profiles.

(2) The relationship between loop alterations and copy-number variations (CNVs) is not explored. If conditions permit, it is recommended that overlay differential loops with SNP/Indel/CNV data to exclude spurious differences arising from structural alterations.

While we have not conducted an in-depth SNP analysis, we have clarified our discussion of the karyotype analysis on pages 21 and 23 and how we mitigated these effects when identifying differential loops between cell lines.

(3) The horizontal and vertical coordinates of the diagram are difficult to view; it is recommended that the size of the text on the picture be adjusted to ensure that it is clear to read. Some of the text coordinates of the figure are labeled in gray; it is recommended that they be in black.

The clarity of the figures has been improved.

**Reviewer #3 (Recommendations for the authors):**

I really like this paper. I think if the cancer focus can be down-emphasized (because I'm not fully clear what we've really learned about cancer), then it represents a nice dataset and a thoughtful, comprehensive analysis.

We greatly appreciate the kind words and helpful feedback. The cancer focus has been toned down throughout the manuscript, as suggested.

Minor Concerns:

(1) The authors present a nice summary of the topological changes across samples. However, summary statistics can mask noise/bias and also don't fully convey the effect size of the reported changes. Highlighting individual loci and visualizing these would strengthen the paper and participate in maintaining a high standard for our genomic studies of topology, in which we summarize, but also provide representative examples. I would appreciate seeing more example plots at distinct loci (even if in the supplemental information).

We have included several more example regions in Supp. Fig. 7 and 12, including four looped genes that change similarly between the MCF10 series and TCGA-BRCA data (2 stably looped genes and 2 differentially looped genes, 2 up-regulated and 2 downregulated), and six differentially looped and differentially expressed genes (3 which change in the same direction as the loops, and 3 which change in the opposite direction).

(2) "To identify loops that changed significantly during cancer progression, we assessed changes in contact frequency among every loop in each cell type, correcting for karyotypic differences that result in differences in coverage between cell lines (see Methods)." The Methods section is not adequately explained. Also, could you go a bit deeper to define if these large-scale changes shift the 3D genome specifically? This is hard, but there may be some low-hanging fruit given the otherwise fairly isogenic features in your model.

We have added more detail to the Methods section on pages 21 and 23 on how karyotypic abnormalities were included in our analysis and differential loop calling. A deeper analysis of how large-scale karyotypic changes affect chromatin organization (i.e. through the formation of neoloops and TADs through translocations) is indeed an attractive subject, but due to its complexity requires a separate dedicated study.

(3) "Approximately half of chromatin loops featured some combination of active gene promoters and enhancers within 10kb of loop anchors". The authors have high-resolution topology data and should be more stringent; these features should have to overlap loop anchors or at least use a distance less than 10kb, which, in some sense, forfeits the advantages of high-resolution topology data.

The threshold of 10kb was chosen for several specific reasons: First, the loop sizes detected here are large enough that this relatively large region still represents a small fraction of the loop span, and these regions are reasonably considered anchor-proximal. Second, the loops we detect are non-punctate, both in aggregate analysis (Figure 1H, bottom) and at individual loci (see example regions), showing increased contact frequency among several 5kb or 10kb bins. Therefore, adding 10kb to either side (2 pixels on 5kb maps and 1 pixel on 10kb maps) ensures that the full region of increased contact frequency is included. Finally, ultra-resolution Hi-C data has also shown that loops remain diffuse even with 1kb resolution maps (albeit they do get smaller than the 30kb used here) (Harris & Gu 2023). We have added a brief justification of this overlap size to the text on page 24.

(4) "These results show that not only changes in either contact frequency and enhancer activity correlate with increased gene expression, but they also correlate with each other, suggesting a potentially linked functional role during enhancer-promoter communication." The authors could use this opportunity to disentangle the contributions of loops and chromatin modifications a bit more. The exceptions are of interest - e.g., loop is stable, gene expression changes or loop changes, gene expression does not. Highlighting exemplar cases for these exceptions (rather than just a genomics summary) would be nice to see.

The additional example regions we have included in Supp. Fig. 7 and 12 now showcase a wider variety of scenarios; in addition to more examples of static loops with gene expression changes (Fig. 2, Supp. Fig. 7E-F) and differential loops with matching gene expression changes (Fig. 4, Supp. Fig. 7C-D, Supp. Fig. 12A-C), we now also feature examples of differential loops where gene expression changes in the opposite direction (i.e. a strengthened loop at a down-regulated gene, Supp. Fig. 12D-F).
